# Toxin expression during *Staphylococcus aureus* infection imprints host immunity to inhibit vaccine efficacy

**DOI:** 10.1038/s41541-022-00598-3

**Published:** 2023-01-24

**Authors:** Omid Teymournejad, Zhaotao Li, Pavani Beesetty, Ching Yang, Christopher P. Montgomery

**Affiliations:** 1 Center for Microbial Pathogenesis, Abigail Wexner Research Institute at Nationwide Children’s Hospital, Columbus, OH US; 2 Department of Pediatrics, The Ohio State University College of Medicine, Columbus, OH US; 3 Division of Critical Care Medicine, Nationwide Children’s Hospital, Columbus, OH US; 4 Present Address: Department of Pathology, College of Medicine, University of Illinois at Chicago, Chicago, IL US; 5 Present Address: Toronto General Hospital Research Institute, University Health Network, Toronto, Ontario Canada; 6 Present Address: Veterinary Biomedical Sciences, College of Veterinary Medicine, Long Island University, Brookville, NY US

**Keywords:** Protein vaccines, Bacteriology

## Abstract

*Staphylococcus aureus* infections are a major public health issue, and a vaccine is urgently needed. Despite a considerable promise in preclinical models, all vaccines tested thus far have failed to protect humans against *S. aureus*. Unlike laboratory mice, humans are exposed to *S. aureus* throughout life. In the current study, we hypothesized that prior exposure to *S. aureus* “imprints” the immune response to inhibit vaccine-mediated protection. We established a mouse model in which *S. aureus* skin and soft tissue infection (SSTI) is followed by vaccination and secondary SSTI. Unlike naïve mice, *S. aureus*-sensitized mice were incompletely protected against secondary SSTI by vaccination with the inactivated α-hemolysin (Hla) mutant Hla_H35L_. Inhibition of protection was specific for the Hla_H35L_ vaccine and required *hla* expression during primary SSTI. Surprisingly, inhibition occurred at the level of vaccine-elicited effector T cells; *hla* expression during primary infection limited the expansion of T cells and dendritic cells and impaired vaccine-specific T cell responses. Importantly, the T cell-stimulating adjuvant CAF01 rescued inhibition and restored vaccine-mediated protection. Together, these findings identify a potential mechanism for the failure of translation of promising *S. aureus* vaccines from mouse models to clinical practice and suggest a path forward to prevent these devastating infections.

## Introduction

Skin and soft tissue infections (SSTI) are a major public health problem with a national economic burden of $15 billion/year^1^. *Staphylococcus aureus* is the most common cause of SSTI, and recurrent infections are common^2,3^. Antibiotic treatment is the mainstay of therapy, but antibiotic-resistant *S. aureus* isolates have emerged^4^. Therefore, preventative strategies are urgently needed. Unfortunately, despite considerable effort, no vaccine is currently licensed to prevent *S. aureus* infections^5^.

It is not clear whether “natural” immune responses against *S. aureus* protect against infection. The high rates of recurrent infection in individuals with SSTI—up to 50% within a year—suggest that protection is incomplete at best^3,6,7^. Given the failures of all vaccine efforts to date^8^, it is imperative to determine the nature of protective immunity against *S. aureus*. Paradoxically, most individuals have detectable levels of *S. aureus*-specific antibodies^9^ and memory T cells^10,11^, consistent with the notion that exposure to *S. aureus* is ubiquitous and persists throughout the lifespan. These findings support the hypothesis that natural exposure to *S. aureus* “imprints” the immune system resulting in resistance to vaccination. Indeed, Tsai et al. recently reported that *S. aureus* infection may imprint non-protective antibody responses that interfere with protective antibodies elicited by vaccination^12^. Natural exposure to pathogens is thought to be a challenge in vaccination against a variety of pathogens. For example, exposure early in life to influenza shapes the immune system in such a way that subsequent responses to vaccination with a heterologous strain are inhibited at the expense of recall of responses against the original strain^13^. Francis called these patterned responses “Original Antigenic Sin”^14^. Whether human exposure to *S. aureus* contributes to the failure of vaccine efforts is not yet clear.

It is also not clear what immunologic mechanisms should be targeted with candidate vaccines. Although there is evidence in murine models that both cellular and humoral immune responses are important for protection against *S. aureus*, human studies suggest that T cells are most important in determining susceptibility to infection^15^. We and others have identified immune responses against the staphylococcal a-hemolysin (Hla) as protective against *S. aureus* SSTI^16,17^. Although Hla-specific antibody responses are clearly important for protection in mouse models, there is also a role for T cell responses^18–22^. We reported that concomitant *S. aureus* SSTI interferes with vaccine-mediated protective antibody and T cell responses in a mouse model by the preferable presentation of immunodominant, but not protective, epitopes in a manner dependent on the host major histocompatibility complex, providing one potential mechanism by which *S. aureus* may thwart vaccine-mediated protection^18^.

In the current study, we sought to understand how prior exposure to *S. aureus* could inhibit vaccine-mediated protection. Using a novel mouse model of *S. aureus* SSTI in which infection “imprints” host immune responses, we found that prior infection inhibits the ability of vaccination to elicit protection against secondary infection. Importantly, this inhibition was dependent on *hla* expression during primary infection and specific to Hla-targeted vaccination. Our findings demonstrate that toxin expression during infection inhibits vaccine-specific T cell-mediated protection against secondary infection and can be overcome by targeting T cell responses using alternative vaccine adjuvants.

## Results

### *S. aureus* SSTI inhibits the efficacy of Hla_H35L_ vaccination against secondary dermonecrosis

Based on our findings that *saeRS* expression during primary SSTI is necessary for protection against recurrent SSTI^23^, we developed vaccines comprised of the *sae*-regulated antigens leukotoxin E (LukE), Panton-Valentine leucocidin S (LukS-PV), serine protease B (SplB), a cysteine protease (SspB), and an α-hemolysin mutant (Hla_H35L_)^18,24^. We reported that the 4S (LukE, LukS-PV, SplB, and SspB), and 5S (4S + Hla_H35L_) vaccines protected against *S. aureus* dermonecrosis^18^; 5S demonstrated superior protection compared with 4S or Hla_H35L_ alone by virtue of eliciting complementary antibody and T cell responses. However, in contrast to experimental infection in naïve mice, most humans have evidence of prior exposure to *S. aureus*. We, therefore, sought to develop a more clinically relevant mouse model in which to test candidate vaccines. Moreover, we hypothesized that if exposure to *S. aureus* inhibited the ability to successfully vaccinate against future infections, such inhibition would be circumvented by the use of a multivalent vaccine. Specifically, we expected that exposure to *S. aureus* would inhibit the efficacy of vaccination with Hla_H35L_ and 4S, but that vaccination with 5S would overcome this inhibition.

**Fig. 1:**
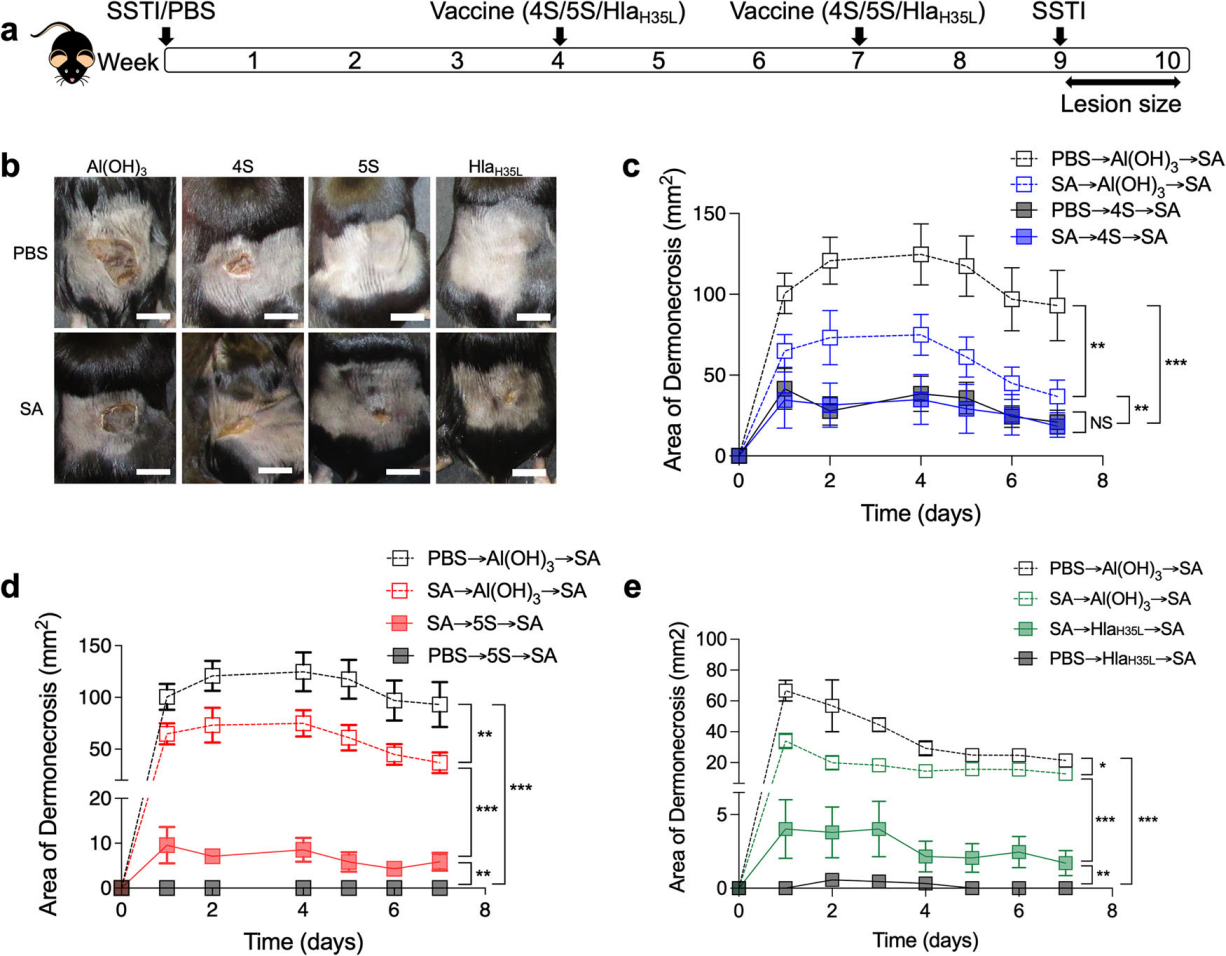
*S. aureus* SSTI interferes with the efficacy of HlaH35L vaccine-mediated protection against dermonecrosis. **a** Experimental model: C57BL/6 mice were infected with *S. aureus* (SA) SSTI followed by vaccination with “4S” (LukE, LukS-PV, SplB, SspB), “5S” (4S + Hla_H35L_), or Hla_H35L_, followed by secondary SSTI. Aluminum hydroxide (Al(OH)_3_) was used as an adjuvant; Al(OH)_3_ group received the adjuvant alone. **b** Representative skin lesions on day 2 post-infection. Scale bar = 10 mm. **c** Prior SSTI had no impact on the efficacy of 4S vaccination. **d** Mice that received SSTI prior to 5S vaccination (SA → 5S → SA) had larger dermonecrotic skin lesions, compared with naïve 5S-vaccinated mice (PBS → 5S → SA). **e** Mice that received SSTI prior to Hla_H35L_ vaccination (SA → Hla_H35L_ → SA) had larger dermonecrotic lesions compared with naïve Hla-vaccinated mice (PBS → Hla_H35L_ → SA). *N* = 5 mice/group; 1 representative experiment of at least 3 repeats is presented. Data are presented as mean ± SEM and were analyzed using two-way ANOVA with repeated measures and Tukey’s post-test. * indicates *p* < 0.05; **p < 0.01; ***p < 0.001; NS not significant.

To test this hypothesis, we established a mouse model in which mice were infected with *S. aureus* (or sham infection with phosphate-buffered saline (PBS)) followed by vaccination with 4S, 5S, or Hla_H35L_ (4 and 7 weeks post-SSTI) and secondary SSTI 2 weeks following vaccination (Fig. 1a). This approach allows for the clearance of bacteria from the skin lesions prior to vaccination. Consistent with our previous report, vaccination with 4S, 5S, and Hla_H35L_ protected naïve mice against SSTI (Fig. 1b–e). We were surprised to find no impact of prior SSTI on the efficacy of 4S vaccination; there were no significant differences in lesion size or colony-forming units (CFUs) during secondary infection of mice that received PBS or SSTI prior to 4S vaccination (Fig. 1b, c and Supplementary Fig. 1). In contrast, whereas naïve 5S-vaccinated mice had no dermonecrosis, SSTI-sensitized 5S-vaccinated mice unexpectedly all had dermonecrotic lesions, although there were no significant differences in bacterial CFUs from the lesions (Fig. 1b, d and Supplementary Fig. 1). This suggested that the ability of SSTI to inhibit vaccine efficacy was specific to Hla-mediated protection. Indeed, there were larger dermonecrotic lesions in SSTI-exposed Hla_H35L_-vaccinated mice during secondary SSTI, compared with naïve Hla_H35L_-vaccinated mice (Fig. 1b, e). Together, these findings suggest that prior SSTI specifically inhibits Hla vaccine-mediated protection.

### *S. aureus* SSTI interferes with Hla_H35L_ vaccine-elicited T cell, but no antibody responses

**Fig. 2:**
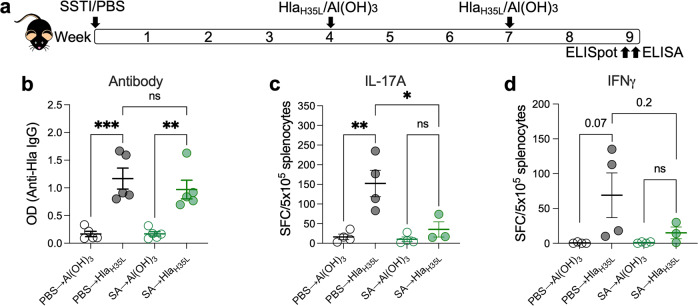
*S. aureus* SSTI impairs vaccine-specific T-cell responses, but not antibody responses. **a** Experimental model: C57BL/6 mice were infected with *S. aureus* (SA) SSTI followed by vaccination with Hla_H35L_ and quantification of anti-Hla IgG levels by ELISA or Hla-specific T cell responses by IL-17A or IFNγ ELISpot on cultured splenocytes. **b** There were no significant differences in the levels of Hla-specific IgG elicited by vaccination, regardless of whether mice received primary SSTI prior to vaccination. **c** Mice that received primary SSTI prior to vaccination with Hla_H35L_ (SA → Hla_H35L_) had fewer Hla-specific IL-17A staining cells following vaccination, compared with naïve vaccinated mice (PBS → Hla_H35L_). **d** Although there was a trend toward fewer Hla IFNγ staining cells in mice that received SSTI prior to vaccination, the differences were not significant. *N* = 3–5 mice/group; one representative experiment of at least two repeats is presented. Data are presented as mean ± SEM and were analyzed using one-way ANOVA with Tukey’s post-test. * indicates *p* < 0.05; **p < 0.01; ***p < 0.001; NS not significant.

Vaccination with Hla_H35L_ elicits antibody-mediated protection against SSTI^25,26^. We, therefore, hypothesized that SSTI inhibits the ability of vaccination to elicit Hla-specific antibodies. To test this, mice were infected with *S. aureus* SSTI or PBS, followed by vaccination with Hla_H35L_ or Al(OH)_3_ alone (Fig. 2a). Anti-Hla antibody levels were quantified by enzyme-linked immunosorbent assay (ELISA) 2 weeks following vaccination. Surprisingly, there were no significant differences in Hla-specific IgG levels among vaccinated mice, regardless of whether they had previously been infected with SSTI (Fig. 2b). This suggested that SSTI did not inhibit Hla vaccine-specific antibody responses.

Th1 and Th17-polarized immune responses also contribute to protection in mouse models of *S. aureus* infection^17,21,22,27^. Therefore, we quantified antigen-specific effector T cell responses by interleukin-17A (IL-17A) and interferon-γ (IFNγ) ELISpot in splenocytes following vaccination. Surprisingly, mice that received Hla_H35L_ vaccination following SSTI had fewer Hla-specific IL-17A staining cells, compared with mice that received PBS prior to vaccination (Fig. 2c). Similarly, SSTI-primed Hla-vaccinated mice had a trend toward fewer Hla-specific IFNγ staining cells, compared with sham-infected Hla-vaccinated mice, although the differences were not significant (Fig. 2d). These findings suggested that exposure to *S. aureus* during SSTI interfered with Hla_H35L_ vaccination through inhibition of vaccine-specific T cell responses, but not antibody responses.

We next sought to determine whether SSTI elicited antibodies that interfered with the ability to respond to vaccination. To test this, serum from convalescent C57BL/6 mice (following *S. aureus* SSTI) was transferred to naïve mice 1 day prior to Hla_H35L_ vaccination, followed 3 weeks later by secondary SSTI (Supplementary Fig. 2A). Passive transfer of serum from *S. aureus*-infected mice did not interfere with the efficacy of Hla_H35L_ vaccination; lesion sizes in mice that received serum from *S. aureus* infected mice prior to vaccination were similar to those received serum from naïve mice (Supplementary Fig. 2B, C). We next tested whether SSTI-elicited T cells could interfere with the ability to respond to vaccination by transfer of T cells from convalescent C57BL/6 mice to naïve mice 1-day prior Hla_H35L_ vaccination, followed by secondary *S. aureus* SSTI (Supplementary Fig. 2D). As we observed with serum, transfer of T cells from *S. aureus*-infected mice did not interfere with the efficacy of Hla_H35L_ vaccination; lesion sizes in mice that received T cells from *S. aureus* infected mice prior to vaccination were similar to those that received serum from naïve mice (Supplementary Fig. 2E, F). These results suggest that infection-elicited antibodies or T cells do not mediate the inhibition of vaccine efficacy.

### Bacterial persistence during primary SSTI is required for the inhibition of vaccine efficacy

**Fig. 3:**
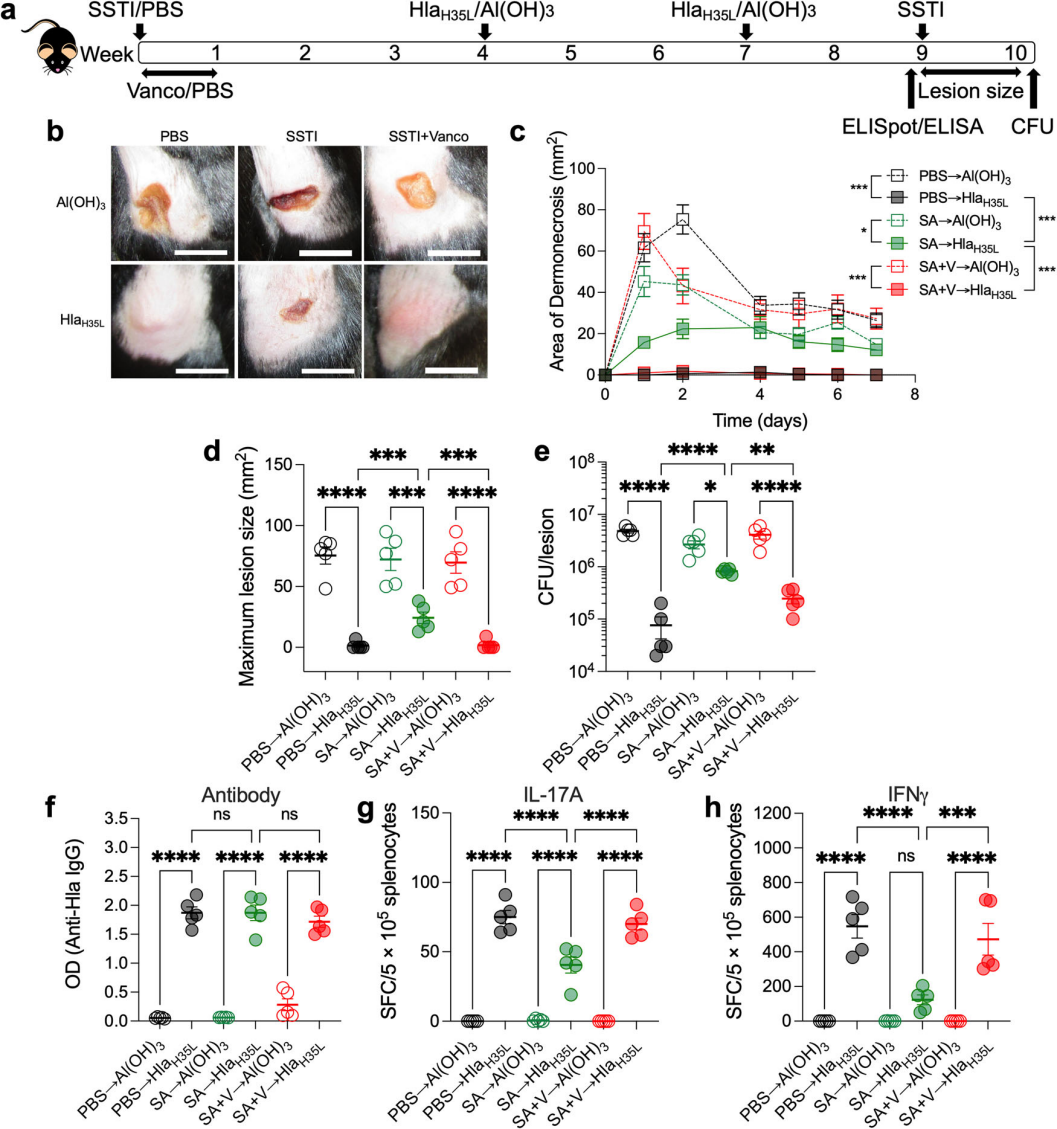
Bacterial persistence during primary SSTI is necessary for impairment of Hla_H35L_ vaccine efficacy and T cell responses. **a** Experimental model: mice were infected with *S. aureus* (SA) SSTI followed by vaccination with Hla_H35L_ and secondary SSTI or quantification of Hla-specific IgG levels and T cell responses. Selected groups were treated with vancomycin during primary SSTI. **b** Representative lesions on day 2. Scale bar = 10 mm. **b**–**d** Protection against secondary dermonecrosis was rescued in mice that received vancomycin during primary SSTI (SA + V → Hla_H35L_), compared with untreated vaccinated mice (SA → Hla_H35L_). **e** Enhanced bacterial clearance in the SA + V → Hla_H35L_ group, compared with the SA → Hla_H35L_ group. **f** Vancomycin treatment did not impact vaccine-elicited Hla-specific IgG levels. **g**, **h** Vancomycin treatment rescued Hla-specific IL-17A (**g**) and IFNγ (**h**) responses following vaccination; there were more IL-17A and IFNγ staining cells in the SA + V → Hla_H35L_ group, compared with the SA → Hla_H35L_ group. *N* = 5 mice/group; one representative experiment of two repeats is presented. Data are presented as mean ± SEM and were analyzed using two-way ANOVA with repeated measures and Tukey’s post-test (**c**) or one-way ANOVA with Tukey’s post-test (**d**–**h**). CFU data were log_10_ transformed for analysis.* indicates *p* < 0.05; **p < 0.01; ***p < 0.001; ****p < 0.0001; NS not significant.

We next tested whether bacterial persistence during primary infection was necessary to inhibit Hla_H35L_ vaccine efficacy. We reported that vancomycin treatment enhanced bacterial clearance and resolved skin lesions^28^. Following primary *S. aureus* SSTI, mice were treated with vancomycin (or PBS) every day for 7 days. Mice were vaccinated with Hla_H35L_ followed by secondary SSTI (Fig. 3a). Mice that were treated with vancomycin following primary SSTI had smaller lesions (Fig. 3b–d) and fewer bacteria in the skin lesions (Fig. 3e) following vaccination, compared with untreated previously infected vaccinated mice. Therefore, treatment of primary SSTI with vancomycin restored Hla_H35L_ vaccine efficacy.

We next tested whether vancomycin treatment would rescue vaccine-specific T cell and antibody responses. Consistent with our previous findings, anti-Hla antibody levels were not different among vaccinated mice, regardless of prior SSTI or vancomycin treatment (Fig. 3f). However, Hla-vaccine-specific IL-17A and IFNγ T cell responses were rescued in mice that received treatment of primary SSTI with vancomycin prior to vaccination; T cell responses were similar to those of naïve vaccinated mice and increased compared with infected untreated mice (Fig. 3g, h). Together, these findings suggest that bacterial persistence during primary SSTI is necessary to inhibit vaccine efficacy and vaccine-specific T-cell responses.

### *Hla* expression during primary SSTI interferes with Hla_H35L_ vaccine-specific T-cell responses and protective efficacy

**Fig. 4:**
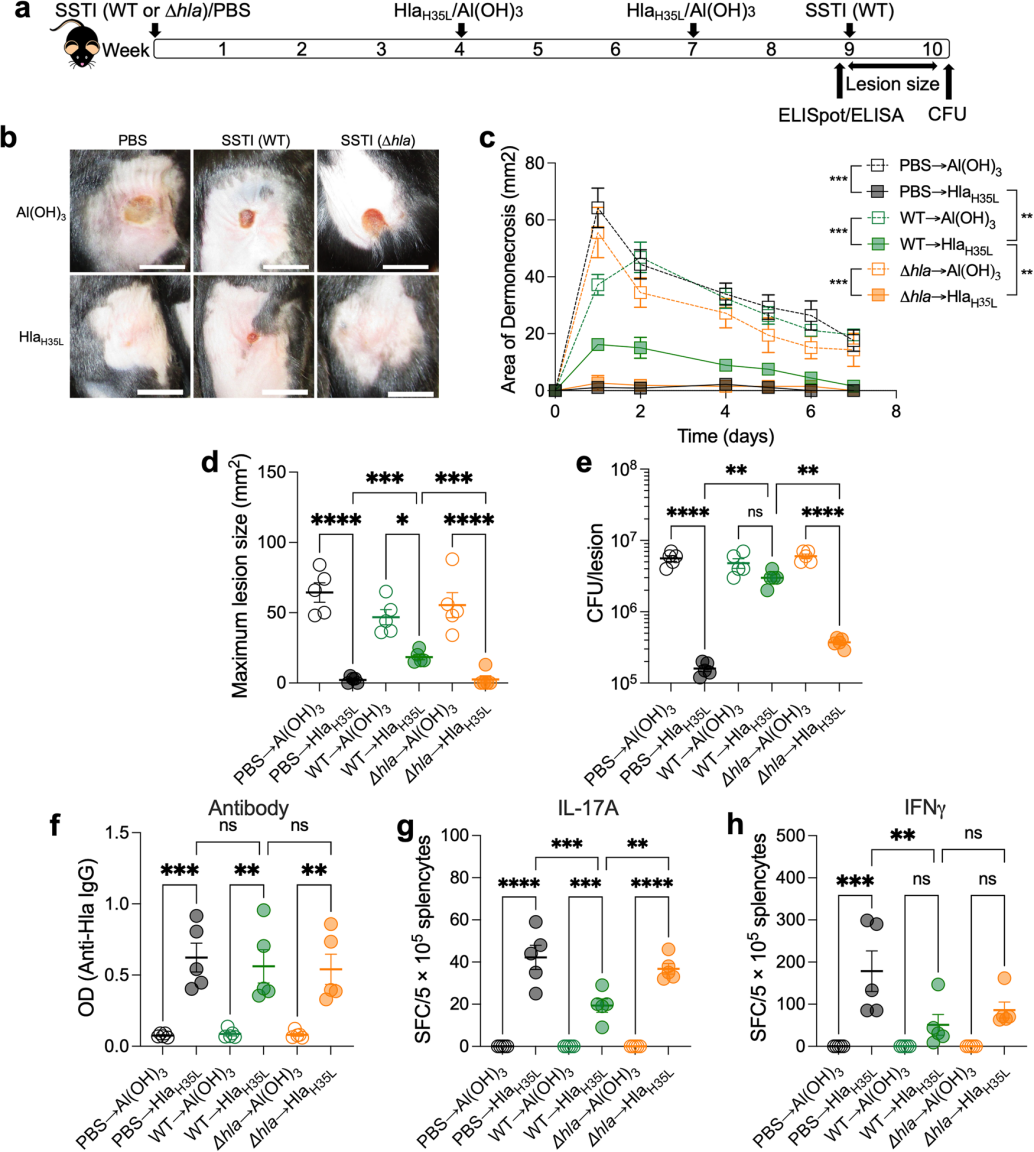
Hla expression during SSTI is necessary for impairment of vaccine efficacy and vaccine-specific T cell responses. **a** Experimental model: C57BL/6 mice were infected with wild-type (WT) *S. aureus* or an isogenic *hla* deletion mutant (*∆hla*) SSTI followed by vaccination at a distal site with Hla_H35L_ and secondary SSTI with WT *S. aureus* or quantification of anti-Hla IgG levels by ELISA or Hla-specific T cell responses by IL-17A or IFNγ ELISpot on cultured splenocytes. **b** Representative photos of mouse lesions on day 2. Scale bar = 10 mm. **b**–**d** Vaccine-mediated protection against secondary dermonecrosis was rescued in mice that received primary SSTI with *∆hla* (*∆hla* → Hla_H35L_), compared with mice that received primary SSTI with WT (WT → Hla_H35L_). **e** Vaccination resulted in enhanced bacterial clearance in the *∆hla* → Hla_H35L_ group, compared with the WT → Hla_H35L_ group. **f** Vaccine-elicited antibody levels were similar, regardless of whether mice received primary infection with WT or *∆hla*. **g** Primary SSTI with *∆hla* rescued Hla-specific IL-17A responses following vaccination; there were higher numbers of IL-17A staining cells in the *∆hla* → Hla_H35L_ group, compared with the WT → Hla_H35L_ group. **h** Although there was a trend toward higher numbers of IFNγ staining cells in the *∆hla* → Hla_H35L_ group, the differences were not significant. *N* = 5 mice/group; 1 representative experiment of at least two repeats is presented. Data are presented as mean ± SEM and were analyzed using two-way ANOVA with repeated measures and Tukey’s post-test (**c**) or one-way ANOVA with Tukey’s post-test (**d**–**h**). CFU data were log_10_ transformed for analysis. * indicates *p* < 0.05; **p < 0.01; ***p < 0.001; ****p < 0.0001; NS not significant.

The requirement for bacterial persistence suggested that expression of a bacterial protein-mediated vaccine inhibition. Lee et al. reported that *hla* expression limits the expansion of local and systemic T cell populations during SSTI^29^. Our findings suggested that inhibition of vaccine efficacy was specific for T cells; we, therefore, hypothesized that Hla inhibited vaccine-elicted T cell responses. We adapted our model by performing primary SSTI with WT or *Δhla*, followed by vaccination with Hla_H35L_ and secondary SSTI with WT *S. aureus* (Fig. 4a). Consistent with our hypothesis, primary SSTI with *Δhla* rendered mice strongly protected by vaccination against dermonecrosis, compared with those that received primary SSTI with WT *S. aureus* (Fig. 4b–d). The reduction in lesion size was accompanied by enhanced bacterial clearance from the skin lesions 7 days after infection (Fig. 4e). Therefore, *hla* expression during primary SSTI inhibited Hla_H35L_ vaccine efficacy.

We next tested whether *hla* expression during primary SSTI inhibits vaccine-specific antibody and T-cell responses. Consistent with our previous findings, anti-Hla IgG levels were not different among vaccinated mice, regardless of whether primary SSTI was performed with WT or *Δhla* (Fig. 4f). However, there were more Hla-vaccine-specific IL-17A staining cells in mice that received primary SSTI with *Δhla* prior to vaccination, compared with those that received WT *S. aureus* prior to vaccination (Fig. 4g). Similarly, there was a trend toward stronger Hla-specific IFNγ responses in mice that were infected with *Δhla* prior to vaccination, but the differences were not significant (Fig. 4h). Together, these findings demonstrate that *hla* expression during primary SSTI inhibits vaccine efficacy and suggests that inhibition is mediated by impaired vaccine-specific T-cell responses.

### *Hla* expression limits the expansion of T cells and DCs

**Fig. 5:**
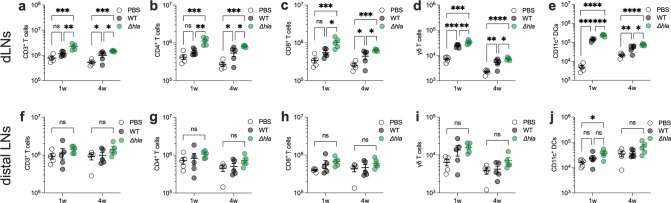
Hla expression during SSTI durably impairs the expansion of T cells and dendritic cells in draining lymph nodes. Following SSTI with wild-type (WT) *S. aureus* or an isogenic *hla* deletion mutant (*∆hla*), local draining lymph nodes (dLN) or distal LN were harvested 1 and 4 weeks after infection, and flow cytometry performed to quantify CD3^+^, CD4^+^, CD8^+^, or γδ T cells and CD11c^+^ dendritic cells (DC). **a**–**e** There were higher numbers of all cell populations tested in local dLNs following infection with *∆hla*, compared with WT. These differences persisted for 4 weeks following the infection. **f**–**j** In contrast, there were no significant differences in the numbers of T cells and DCs in distal LNs 1 or 4 weeks following infection between mice infected with WT or *∆hla*. *N* = 5 mice/group; one representative experiment of at least two repeats is presented. Data are presented as mean ± SEM and were analyzed using one-way ANOVA on log_10_-transformed values with Tukey’s post-test. * indicates *p* < 0.05; **p < 0.01; ***p < 0.001; ****p < 0.0001; NS not significant.

Our findings and those of Lee et al.^29^ suggested that *hla* expression during SSTI mediates inhibition of vaccine efficacy by limiting the expansion of potentially vaccine-responsive T cells. We, therefore, quantified T cell and dendritic cell (DC) populations by flow cytometry in draining and distal LNs 1 or 4 weeks following SSTI with WT *S. aureus* or an isogenic *hla* deletion mutant (*Δhla*) (gating strategy, Supplementary Fig. 3). Consistent with their findings and a role for Hla in limiting the expansion of immune cells, we found that the total number of CD3^+^, CD4^+^, CD8^+^, γδ T cells, and CD11c^+^ DCs 1 week after infection were higher in draining LNs in the mice infected with *Δhla*, compared with WT (Fig. 5a–e). However, these differences were limited to locally draining LN, because there were no significant differences in these cell populations in distal LNs (Fig. 5f–j). Importantly, the differences in APC and T cell populations in draining LNs persisted for at least 4 weeks after infection (i.e., the time of vaccination) (Fig. 5a–e), but there remained no differences in distal LNs (Fig. 5f–j). To better understand the cytokine profiles of the impacted T cells, we quantified IL-17A^+^ and IFNγ^+^ CD4^+^, CD8^+^, and γδ T cells after 1 week in dLNs. We found more IL-17A^+^ and IFN-γ^+^ CD4^+^ T cells after 1 week in dLNs in the mice group that were infected with *Δhla*, compared with WT (Supplementary Fig. 4A). There were also higher numbers of IFNγ^+^ CD8^+^ and γδ T cells following infection with *Δhla*, compared with WT, but no differences in IL-17A^+^ CD8^+^ or γδ T cells (Supplementary Fig. 4B, C). To determine whether the preferential expansion of immunosuppressive regulatory T cells (T_regs_) might contribute to vaccine resistance, we quantified Foxp3^+^ CD4^+^ T cells after 1 and 4 weeks in dLNs and distal LNs. However, as we observed with other T cell subsets, there were higher numbers of Foxp3^+^ CD4^+^ T cells after 1 and 4 weeks in dLNs in the mice group that were infected with *Δhla*, compared with WT (Supplementary Fig. 4D). There were also no significant differences in the numbers of Foxp3^+^ CD4^+^ T cells in distal LNs, regardless of the infecting isolate (Supplementary Fig. 4E).

To confirm that Hla specifically mediated impairment of T cell expansion, we next transferred Hla-specific antiserum to naïve mice 1 day before SSTI with WT *S. aureus* followed by quantification of T cell populations 1 week after infection (Supplementary Fig. 5A). In support of a role for Hla in impairing expansion of T cell populations, there were higher numbers of CD3^+^, CD4^+^, CD8^+^, and γδ T cells in dLNs of mice that received Hla-specific antiserum prior to infection, compared with those that received naïve serum (Supplementary Fig. 5B–E). Consistent with our results with WT vs. *Δhla*, there were more IL-17^+^ CD4^+^ T cells in mice that received Hla-specific antiserum prior to infection, compared with those that received naïve serum (Supplementary Fig. 6A), but there were no significant differences in other IL-17^+^ or IFNγ^+^ T cell populations (Supplementary Fig. 6B, C). These results confirmed that antibody-mediated neutralization of Hla rescued T cell expansion during SSTI. Finally, to confirm that Hla interacted with its cellular receptor ADAM10 (A Disintegrin and metalloproteinase domain-containing protein 10)^30–32^ to drive the toxin-mediated impaired expansion of T cells, mice received the ADAM10 inhibitor (GI254023X) 1 h before SSTI (Supplementary Fig. 5A). Consistent with our previous findings, we found higher numbers of CD3^+^, CD4^+^, CD8^+^, and γδ T cells in dLNs 1 week after infection in mice that received ADAM10i prior to infection, compared with those that received vehicle (DMSO) alone (Supplementary Fig. 5F–I). However, there were no significant differences in IL-17^+^ or IFNγ^+^ T cell populations among these groups (Supplementary Fig. 6D–F). Taken together, these findings demonstrate that expression of *hla* during primary SSTI limits the expansion of T cells and DCs, suggesting a potential mechanism by which infection impairs the ability to respond to subsequent vaccination.

### T cell responses contribute to Hla_H35L_ vaccine-mediated protection

**Fig. 6:**
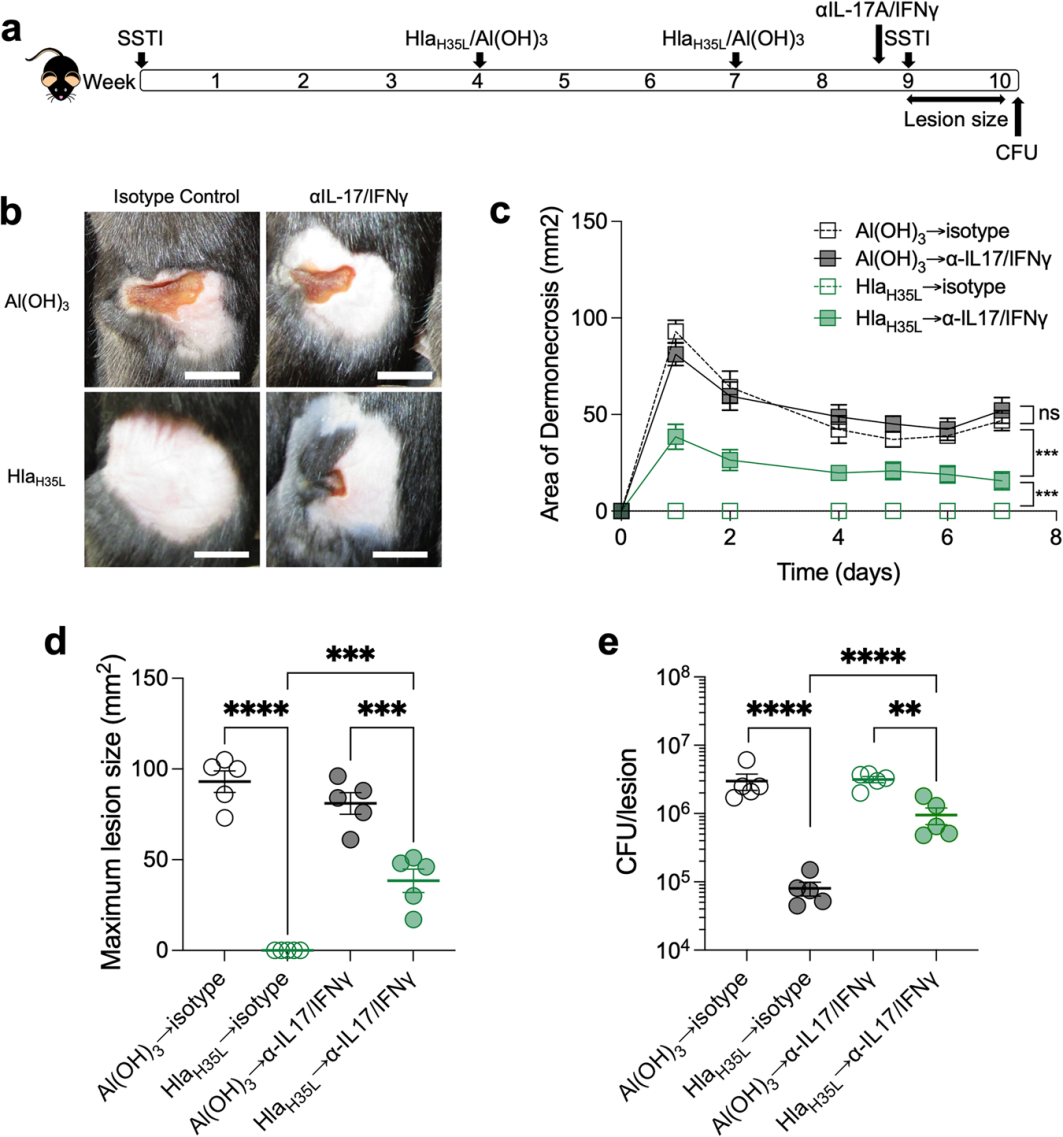
IL-17A and IFNγ T cell responses contribute to Hla_H35L_ vaccine-mediated protection. **a** Experimental model: C57BL/6 mice were infected with *S. aureus* SSTI followed by vaccination with Hla_H35L_ and secondary SSTI. Prior to secondary SSTI, groups of mice were treated with IL-17A and IFNγ neutralizing antibodies or isotype control antibodies. **b** Representative photos of mouse lesions on day 2. Scale bar = 10 mm. **b**–**d** Neutralization of IL-17A and IFNγ impaired vaccine-mediated protection against secondary dermonecrosis (Hla_H35L_ → αIL17/IFNγ), compared with mice that received isotype control antibodies (Hla_H35L_→isotype). **e** Neutralization of IL-17A and IFNγ also impaired vaccine-mediated bacterial clearance. *N* = 5 mice/group; one representative experiment of two repeats is presented. Data are presented as mean ± SEM and were analyzed using two-way ANOVA with repeated measures and Tukey’s post-test (**c**) or 1-way ANOVA with Tukey’s post-test (**d**, **e**). CFU data were log_10_ transformed for analysis. * indicates *p* < 0.05; **p < 0.01; ***p < 0.001; ****p < 0.0001; NS not significant.

We and others have reported that Hla_H35L_ elicits antibody-mediated protection^18,26^. However, our results suggested that *hla* expression during primary SSTI mediates vaccine resistance by inhibiting vaccine-specific IL-17A and IFNγ T cell responses. To confirm the importance of IL-17A and IFNγ in mediating vaccine-elicited protection against dermonecrosis, vaccinated mice were treated with IL-17A and IFNγ neutralizing antibodies prior to secondary SSTI (Fig. 6a). Surprisingly, there were no significant differences in lesion size or bacterial clearance from the skin lesions between sham-vaccinated mice that received isotype control antibodies or αIL-17A/IFNγ antibodies (Fig. 6b–e). However, neutralization of IL-17A and IFNγ in Hla-vaccinated mice resulted in larger dermonecrotic skin lesions, compared with vaccinated mice that received isotype control antibodies (Fig. 6b–d). Similarly, neutralization of IL-17A and IFNγ resulted in decreased bacterial clearance from the skin lesions (Fig. 6e). Therefore, IL-17A and IFNγ were important in mediated vaccine-elicited protection against dermonecrosis and complemented antibody-mediated protection.

### The T cell-stimulating adjuvant CAF01 restores Hla_H35L_ vaccine efficacy and T cell responses

**Fig. 7:**
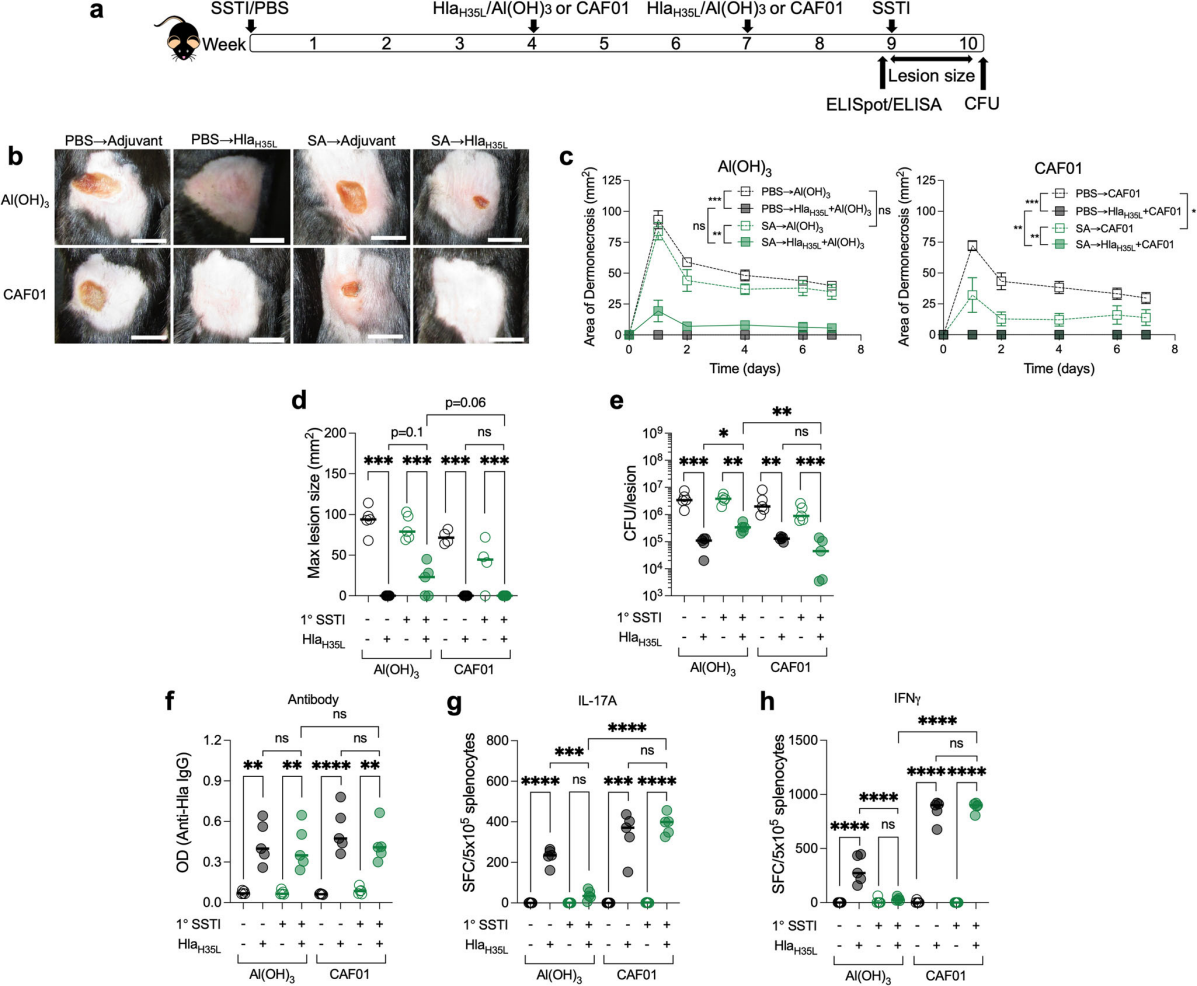
The T cell-stimulating CAF01 adjuvant restores Hla_H35L_ vaccine efficacy and T cell responses in *S. aureus*-sensitized mice. **a** Experimental model: C57BL/6 mice were infected with wild-type (WT) *S. aureus* or an isogenic *hla* deletion mutant (*∆hla*) SSTI followed by vaccination with Hla_H35L_ adjuvanted with Al(OH)_3_ or CAF01. Vaccination was followed by secondary SSTI with WT *S. aureus* or quantification of anti-Hla IgG levels by ELISA or Hla-specific T cell responses by IL-17A or IFNγ ELISpot on cultured splenocytes. **b** Representative photos of mouse lesions on day 2. Scale bar = 10 mm. **b**–**d** Whereas both Al(OH)_3_/Hla_H35L_ and CAF01/Hla_H35L_ protected naïve mice against dermonecrosis, CAF01/Hla_H35L_ vaccination resulted in smaller skin lesions in previously infected mice (SA → Hla_H35L_ + CAF01), compared with Al(OH)_3_/Hla_H35L_ (SA → Hla_H35L_ + Al(OH)_3_). CAF01 alone (SA → CAF01) partially rescued protection in previously infected mice. **e** Vaccination resulted in enhanced bacterial clearance in the SA → Hla_H35L_ + CAF01 group, compared with the SA → Hla_H35L_ + Al(OH)_3_ group. **f** Vaccine-elicited antibody levels were similar, regardless of the adjuvant or whether mice received SSTI prior to vaccination. **g**, **h** Vaccination with CAF01 rescued Hla-specific IL-17A (**g**) and IFNγ (**h**) responses in previously infected mice following vaccination; there were higher numbers of IL-17A staining cells in the SA → Hla_H35L_ + CAF01 group, compared with the SA → Hla_H35L_ + Al(OH)_3_ group. *N* = 5 mice/group; 1 representative experiment of two repeats is presented. Data are presented as mean ± SEM and were analyzed using two-way ANOVA with repeated measures and Tukey’s post-test (**c**) or one-way ANOVA with Tukey’s post-test (**D**–**H**). CFU data were log_10_ transformed for analysis. * indicates *p* < 0.05; **p < 0.01; ***p < 0.001; ****p < 0.0001; NS not significant.

Taken together, our findings suggested that *hla* expression during primary SSTI patterns immune responses, thereby inhibiting vaccine responsiveness by impairing the ability to generate protective vaccine-specific T-cell responses. Because Al(OH)_3_ elicits relatively weak T cell responses^33^, we hypothesized that the use of a potent T cell-stimulating adjuvant would circumvent this inhibition. To test this, we used Cationic Adjuvant Formulation (CAF01) as an adjuvant because it potently elicits Th1/Th17 responses^34^. The CAF platform consists of the quaternary ammonium surfactant *N,N-*dimethyl*-N,N-*dioctadecylammonium (DDA) formulated into liposomes and TBD is inserted into DDA bilayers. We modified our model by vaccinating previously infected mice with Al(OH)_3_ or CAF01-adjuvanted Hla_H35L_, followed by secondary SSTI (Fig. 7a). In naïve mice, there were no significant differences in protection elicited by Hla_H35L_ vaccination using Al(OH)_3_ or CAF01, as assessed by lesion size (Fig. 7b–d) or bacterial clearance from the skin lesions on day 7 (Fig. 7e). However, consistent with our hypothesis, vaccination with CAF01/Hla_H35L_ resulted in superior protection against dermonecrosis in mice that were previously infected, compared with Al(OH)_3_/Hla_H35L_ (Fig. 7b–d). Protection against dermonecrosis was accompanied by enhanced bacterial clearance from the skin lesions in previously infected CAF01/Hla_H35L_ vaccinated mice, compared with Al(OH)_3_/Hla_H35L_ vaccinated mice (Fig. 7e). Interestingly, CAF01 alone rescued protection in previously infected mice, albeit not as strongly as CAF01/Hla_H35L_ (Fig. 7b–e). This argues that a T cell-polarized adjuvant may be beneficial even in the absence of Hla-specific responses.

Next, we tested whether the superior protection elicited using CAF01 as an adjuvant was accompanied by stronger vaccine-specific antibody or T-cell responses. Consistent with our previous findings, there were no significant differences in anti-Hla IgG levels following vaccination, regardless of adjuvant or whether mice had been previously infected prior to vaccination (Fig. 7f). However, consistent with CAF01 eliciting stronger T cell responses, vaccination with of naïve mice with CAF01/Hla_H35L_ elicited more Hla-specific IL-17A and IFNγ staining cells, compared with Al(OH)_3_/Hla_H35L_ (Fig. 7g, h). Importantly, consistent with the superior protection observed, vaccination of previously infected mice with CAF01/Hla_H35L_ elicited dramatically more Hla-specific IL-17A and IFNγ staining cells than did vaccination with Al(OH)_3_/Hla_H35L_ (Fig. 7g, h). Taken together, these findings demonstrate that infection-elicited inhibition of vaccine efficacy and vaccine-specific T cell responses can be circumvented by the use of the potent T cell-stimulating adjuvant CAF01.

## Discussion

Despite a recognition of the importance of *S. aureus* infections and considerable effort, there remains no licensed vaccine. A potential reason for the failure to translate vaccines that successfully protect in experimental models to clinical practice may be the immune history of the vaccine recipient; vaccines are tested in naïve mice before application in humans who invariably have evidence of immune responses against *S. aureus*. In this study, we established a tractable mouse model in which mice are infected with *S. aureus* prior to vaccination. Unlike naïve mice, *S. aureus*-sensitized (previously infected) mice are incompletely protected against secondary SSTI following vaccination with the inactivated Hla mutant Hla_H35L_. Inhibition of protection was specific for the Hla_H35L_ vaccine and required *hla* expression during primary SSTI. Surprisingly, inhibition occurred at the level of vaccine-elicited effector T cells; antibody levels were not impacted. *Hla* expression during primary infection impaired the expansion of T cells and DCs, a major antigen-presenting cell responsible for driving memory T cell generation. Optimal vaccine-mediated protection required robust T-cell responses, and the use of the T-cell-stimulating adjuvant CAF01 circumvented the inhibition of vaccine efficacy elicited by prior infection. Together, these results demonstrate that prior immune history is an important driver of vaccine efficacy and reveal mechanisms by which patterned immune responses can inhibit vaccine responsiveness. Importantly, they also suggest a path toward overcoming patterned immune responses by polarizing vaccine-specific immune responses toward the Th1/Th17 pathways.

We found that *hla* expression during primary infection drives interference with vaccine efficacy. Our findings that *hla* expression impaired the expansion of T cells and DCs in dLNs during primary SSTI (within 7 days) are in agreement with Lee et al., who reported that SSTI with *Δhla* resulted in greater expansion of antigen-specific memory T cells and DCs 4–7 days post-infection^29^. Based on these findings, it is tempting to speculate that Hla directly kills T cells and APCs leading to a reduction in potentially vaccine-responsive APCs and T cells. This hypothesis is supported by several reports demonstrating that Hla kills APCs and T cells^30,35,36^. The specificity of Hla in mediating impairment of T cell responses is further supported by our findings that administration of anti-Hla antiserum and chemical inhibition of the Hla receptor ADAM10 rescued T cell numbers in the dLN. The current study extends these findings by demonstrating that the decreased numbers of T cells and DCs in dLNs persist for at least one month following SSTI, suggesting that toxin-patterned inhibition of memory T cell responses is durable beyond recovery from infection. Our study also demonstrates that Hla-mediated impairment of T cells and DC expansion may have long-term consequences in determining vaccine responsiveness. Interestingly, however, there were no differences in APC or T cell numbers in LNs distant from the site of infection, including the dLNs proximal to the site of vaccination. Thus, at the time of vaccination, there are decreased numbers of DCs and T cells in local dLNs, but not those distant from the site of infection, in vaccine-resistant mice. Therefore, it is not clear that the depletion of vaccine-responsive APCs or T cells is the mechanism that is driving vaccine inhibition. Future studies will reconcile the difference between local and distant T cell memory compartments and will seek to identify the location and phenotype of vaccine-responsive APCs/T cells. An alternative possibility that might explain the ability of SSTI to inhibit vaccine efficacy would be an expansion of an immunosuppressive immune cell population. For example, regulatory T cells (T_reg_) constrain inflammatory T cell responses^37^. However, we found that T_reg_ expansion was similarly impaired following infection with WT *S. aureus* and rescued after infection with *Δhla*. *S. aureus* infection has also been reported to expand a population of immunosuppressive cells called myeloid-derived suppressor cells (MDSC);^38^ therefore, future studies will determine whether the expansion of MDSCs in our model might contribute to the inhibition of vaccine responsiveness.

T cell-mediated immunity is a critical determinant of protection against *S. aureus* infections in humans because individuals with defects in specific T cell pathways are at high risk of *S. aureus* infections. For example, patients with hyper-immunoglobulin E syndrome, in which there are defects in IL-17-mediated defense in the skin and lung, are highly susceptible to recurrent mucocutaneous *S. aureus* infection^15,39^. Similarly, patients with poorly controlled Human Immunodeficiency Virus infection and CD4^+^ T cell lymphopenia have high rates of *S. aureus* infections^40,41^, although it is not clear that this is specifically due to impaired T cell immunity. In contrast, individuals with B cell deficiencies do not appear to be at increased risk of *S. aureus* infection^42–44^. Importantly, all antibody-based vaccination strategies have failed in clinical trials^45–49^, although the reasons for these failures remain unclear. In contrast to human studies, many reports have documented that antibodies and T cells are each important in defense against *S. aureus* infection in mouse models^17,20–22,29^. Therefore, our findings that SSTI only interfered with vaccine-specific T cell responses, but not antibody responses, suggest that this model may be more applicable to human infection. Specifically, vaccination provided partial protection even to *S. aureus*-sensitized mice. We propose that the more modest protection observed in these mice is mediated by vaccine-elicited antibodies, but the ability of naïve mice to respond to vaccination with both strong antibody and T-cell responses resulted in superior protection. It should be noted that we quantified Hla-specific IgG levels; it is, therefore, possible that the vaccine-specific antibodies were functionally different in naïve and experienced vaccinated mice. Future studies will address the epitope-specificity and function of vaccine-elicited antibodies to confirm that primary infection does not interfere with humoral immune responses. This is of particular importance because Tsai et al. recently reported that prior *S. aureus* infection inhibits the efficacy of vaccination with IsdB in mouse models^12^. In their study, they found that inhibition was mediated by the recall by vaccination of non-protective IsdB-specific antibodies originally elicited by infection. Therefore, it is apparent that *S. aureus* has evolved multiple mechanisms by which exposure early in life may inhibit the ability to vaccinate later in life. Taken together, these studies suggest that the use of mouse models of vaccination in experienced mice, and the associated immune response, may better recapitulate human infection.

The Th17/IL-17A pathway is important in defense against *S. aureus* infections, particularly SSTI^17,21,39,50^. It should be noted that γδ T cells are also a potential source of IL-17^21,51,52^, but our studies did not address their role in mediating protection. The magnitude of the IL-17A response may help determine the fate of infection; high levels of IL-17A promote clearance of *S. aureus* SSTI^17,53^. Along these lines, *hla* expression during *S. aureus* SSTI may limit IL-17A secretion, and infection with *Δhla* promotes the rapid expansion of Th1 and Th17 cells^54^. Similarly, there is emerging evidence that the Th1/IFNγ pathway is also important in defense against *S. aureus* infection^22^. While the mechanisms of Th17 and Th1-mediated protection against *S. aureus* infection have not been fully elucidated, it is likely that they work in concert to potentiate neutrophil and macrophage-mediated proinflammatory bacterial clearance^55,56^. In the current study, we found that IL-17^+^ and IFNγ^+^ T cells (mainly CD4^+^) are expanded in higher numbers following infection with *Δhla*, compared with WT. We also found that vaccination with Hla_H35L_ elicited IL-17^+^ and IFNγ^+^ T cells. However, we found that *hla* expression during primary SSTI interfered with the expansion of vaccine-specific IL-17^+^ T cells, and, to a lesser extent, IFNγ^+^ T cells. Importantly, our findings that neutralization of IL-17A and IFNγ inhibits vaccine-mediated protection support a role for targeting both cytokines to achieve optimal vaccine efficacy, particularly in *S. aureus*-experienced individuals. The use of T cell-targeted vaccine adjuvants such as CAF01 is one promising approach to accomplish this.

Adjuvants are used in vaccines to enhance immunogenicity but can also be leveraged to polarize the immune response toward or away from desired antibody and T cell pathways^57^. We demonstrated that “reactivating” the T cell response with the Th1/Th17 stimulating adjuvant CAF01 can polarize the host immune response toward IFNγ and IL-17A and restore vaccine efficacy in *S. aureus*-experienced mice. CAF01 has been tested in vaccines against tuberculosis, HIV, and malaria^58–61^. Indeed, CAF01 rescued vaccine efficacy in *S. aureus*-experienced mice by virtue of enhanced vaccine-specific T-cell responses. Interestingly, CAF01 alone partially rescued protection in previously infected mice. The mechanisms underlying this protection are not clear, but possibilities include restored Hla-independent T-cell responses or augmentation of trained immunity by CAF01^62^; future studies will address this “non-specific” protection. These results are particularly important in light of the fact that most of the *S. aureus* vaccines used in clinical trials are considered self-adjuvanted due to pathogen-associated molecular patterns in the vaccines. For example, V710, StaphVAX, and SA4Ag were unadjuvanted^5,8^. V710 failed to prevent postoperative *S. aureus* infections and was associated with increased mortality in patients who developed *S. aureus* infection^48^. Importantly, mortality was associated with low endogenous levels of IL-17 in patients before receiving the vaccine^63^. Together with the findings of Tsai et al^12^, it is likely that several mechanisms may contribute to the difficulty in vaccinating previously exposed individuals. Specifically, prior exposure may both (i) imprint non-protective antibodies that may be recalled upon vaccination, and (ii) establish non-protective T cell memory that interferes with vaccine-elicited protective T cell responses. Our results suggest that the use of a T cell-stimulating adjuvant such as CAF01 to re-activate the suppressed T cell response could be combined with protective epitope-specific approaches to optimize vaccine efficacy in *S. aureus*-experienced individuals. Importantly, while we agree with Lee et al. that toxin-imprinted immune impairment may justify leveraging the childhood vaccine infrastructure to ensure population-wide immunity^29^, our studies suggest that an alternative targeted approach using novel adjuvants may also enhance protection in older individuals with a lifetime of exposure to *S. aureus*.

There are several important limitations to this study. First, it remains unclear if mouse models reflect the pathogenesis and associated immune response in human *S. aureus* infections. We propose that the use of *S. aureus*-experienced mice better recapitulates human infection, but further confirmatory studies are necessary. Second, the mechanisms by which *hla* expression during primary SSTI suppresses vaccine-specific IL-17, and IFN-γ T cell responses are not entirely clear. We found that *hla* expression durably reduces the number of DCs and CD4^+^, CD8^+^, and γδ T cells in dLNs during and following infection, but it is not clear how these local effects impact systemic vaccine responsiveness. Third, these findings are limited to the C57BL/6 genetic background; future studies will be necessary to determine the impact of host genetics on these findings. Finally, these studies were performed using our SSTI model. Therefore, it is not clear if these findings are generalizable to other infectious syndromes, such as pneumonia or bacteremia.

In conclusion, we developed a mouse model in which *S. aureus* infection “imprints” host immune responses to inhibit the efficacy of subsequent vaccination. These findings highlight the importance of toxin expression in the evasion of protective immunity, specifically protective T-cell responses. This inhibition was circumvented by the use of a T cell-specific adjuvant. Together, these findings suggest that understanding the mechanisms of *S. aureus*-mediated immune evasion can advance vaccine efforts.

## Methods

### Bacteria

*S. aureus* isolates 923 (USA300 isolated from a patient with SSTI), and an isogenic *hla* deletion mutant (*∆hla*) have been previously reported^23^. Bacteria were revived and cultured on tryptic soy agar overnight at 37 °C. The following day, one colony was transferred into tryptic soy broth (TSB) and cultured in a shaking incubator (250 rpm) overnight at 37 °C. On the day of inoculation, the overnight cultures were diluted 1:100 in fresh TSB and cultured at 37 °C for 3 h (approximate optical density at 600 nm [OD_600_] of 1.8). The bacteria were washed in sterile PBS and adjusted to 2 × 10^7^ CFU/50 µl PBS.

### Mice

All animal experiments were approved by the Institutional Animal Care and Use Committee (IACUC) at the Abigail Wexner Research Institute at Nationwide Children’s Hospital (protocol no. AR17-00072) and adhered to the standards of NIH Guide for the Care and Use of Laboratory Animals. For all experiments, female C57BL/6 mice were purchased from Taconic. Primary infection or vaccination was performed when the mice were 7–8 weeks old.

### Mouse model of *S. aureus* SSTI

Prior to inoculation, mice were sedated with isoflurane, and their flanks were shaved with a fine hair clipper and cleaned with Nair™ hair removal cream and ethanol. Mice were subcutaneously inoculated with 2 × 10^7^ CFU of WT or ∆*hla S. aureus* in a volume of 50 µl PBS^17^. Mice were observed to awaken and be given access to food and water throughout the experiment. Mice were treated with 150 mg/kg vancomycin (Medline) or PBS via intraperitoneal injection 3 h post-primary infection, and treatment was continued every day for 7 days. The secondary infection was performed on the opposite flank 7 weeks following the primary infection (2 weeks after the second dose of vaccination). To neutralize Hla or its receptor, mice were treated with 200 µl Hla-specific antiserum retro-orbitally 1 day before SSTI or 20 µM GI254023X (ADAM10 inhibitor) (Sigma-Aldrich) subcutaneously at the infection site, 3 h before SSTI. To assess the severity of the skin infection, lesions were photographed daily for 7 days. Lesion sizes were calculated digitally and compared with a 100 mm^2^ standard. To quantify the lesion bacterial burden, mice were euthanized 7 days after SSTI, and lesions were aseptically dissected and homogenized in PBS. Serial dilutions of the homogenate were plated on mannitol salt agar for colony-forming unit enumeration.

### Vaccination and cytokine neutralization

Our methods for protein purification and vaccination have been reported^18^. Briefly, *lukE*, *splB*, and *ssB* were amplified by a polymerase chain reaction and cloned in pET28a (Novagen). The resulting plasmid was expressed in *Escherichia coli* (DE3, BL21; Invitrogen). The proteins were purified with chromatography using a His-Bind kit (Novagen). Plasmids for *hla*_*H35L*_ and *lukS-PV* purification were generously provided by Juliane Bubeck Wardenburg (Washington University, St. Louis). *hla*_*H35L*_ was cloned in pET24b and purified from *E. coli* (BL21)^64^. *lukS-PV* was cloned in pGEX and purified from *E. coli* (BL21)^64^. Endotoxin was removed using an endotoxin removal kit (Sigma). Mice were vaccinated with one of three vaccines (10 µg of each protein): Hla_H35L_, 4S (LukE, LukS-PV, SplB, and SspB), or 5S (4S + Hla_H35L_). The protein concentration was measured by Bio-Rad protein assay dye reagent concentrate (Bio-Rad). The vaccines were adjuvanted with Al(OH)_3_ (Alhydrogel; Brenntag) or CAF01 ((N,N′-dimethyl-N,N′-dioctadecylammonium bromide (DDA) (Sigma) and α,α′-trehalose-6,6′-dibehenate (TDB) (Invivogen))^65^ at a final concentration of 0.1% or the ratio 5:1, DDA to TDB in a total volume of 200 µl. First and second doses of vaccinations were administered subcutaneously distal from the infection site (between the shoulder blades at the base of the neck) 3 and 5 weeks after the primary infection. For cytokine neutralization, mice were treated intraperitoneally with 500 µg anti-IL-17A (clone 17F3, catalog number BP0173) and anti-IFN-γ (clone XMG1.2, catalog number BP0055) or isotype controls (clone MOPC-21; catalog number BP0083, HRPN; catalog number PB0088, BioXcell) one day prior to SSTI.

### Adoptive transfer

Mice were euthanized 8 weeks after secondary SSTI or PBS by CO_2_ inhalation, and sera and spleens were harvested. T cells were isolated from the single-cell suspension of spleens by negative selection using the Pan T cell Isolation Kit II (1:5; Miltenyi Biotec, catalog number 130-095-130). Briefly, non-target cells were labeled by using a cocktail of biotin-conjugated antibodies against CD14, CD15, CD16, CD19, CD34, CD36, CD56, CD123, and CD235a. Then, non-target cells were magnetically labeled with anti-Biotin microbeads and purified in a magnetic field. Mouse blood was collected by cardiac puncture, and sera were harvested by centrifugation of mouse whole blood. Totally, 200 µl serum or T cells (9 × 10^6^ cells) were transferred retro-orbitally to each recipient mouse 1 day prior to vaccination.

### Quantification of antibody responses

Ninety-six-well ELISA plates (Costar, Corning Inc.) were coated with purified Hla_H35L_ (5 µg/ml). Mouse serum was prepared from whole blood using serum separator tubes (BD Biosciences) and diluted 1:100 in PBS, following which serum was added to the wells. Detection of antigen-specific IgG was performed using alkaline phosphatase (AP)-conjugated goat anti-mouse IgG (1:5000; AffiniPure, Jackson ImmunoResearch, catalog number 115-055-003) and AP substrate p-nitrophenyl phosphate (Sigma-Aldrich) following the manufacturer’s recommendations. Absorbance was measured using a GENios spectrophotometer (Tecan).

### Quantification of T cell responses by ELISpot

Enzyme-linked immunosorbent spot (ELISpot) 96-well plates were coated with anti–IL-17 or anti–IFN-γ antibody (1:500; Becton Dickinson Biosciences, catalog numbers 551309, 555068) overnight at 4 °C. Splenocytes were harvested from mice and plated at 8 × 10^5^ or 4 × 10^5^/well for IL-17 or IFN-γ detection, respectively. The splenocytes were incubated with purified Hla_H35L_ (10 µg/ml) for 24 h at 37 °C with 5% CO_2_. Following washing, biotin-labeled IFN-γ and IL17 detection antibodies (1:500; Becton Dickinson Biosciences, catalog numbers 555067, 551506)were added to the wells, followed by horseradish peroxidase-conjugated anti-biotin (1:250; eBioscience, catalog number 18-41-00-51). Spots were counted following the addition of the substrate solution (BD Biosciences) using an ImmunoSpot series 1 analyzer (Cellular Technology).

### Quantification of T cell responses by flow cytometry

Lymphocytes were isolated from draining lymph nodes following SSTI and processed into single-cell suspensions. Cells were stained for flow cytometry using AquaFluor LiveDead (Life Technologies) solution to exclude dead cells. Two different panels were used for surface and intracellular staining. For surface staining, antibodies against CD8 PerCP Cy 5.5 (0.12 µg, clone:53-6.7, Biolegend, catalog number 100734), CD3 FITC (0.25 µg, clone:145-2C11, Invitrogen, catalog number 11-0031-95), CD4 BUV395 (0.12 µg, clone: GK1.5, BD, catalog number 563790), CD11c eflur450 (0.06 µg, clone: N418, Invitrogen, catalog number 48-0114-82), and γδ TCR PE (0.06 µg, clone: GL3, Biolegend, catalog number 118108) were used. Cells were incubated with antibodies for 30 min on ice in the dark. For intracellular staining, cells were fixed and permeabilized using a Fixing/Permeabilization solution for 30 min in the dark at room temperature, following two subsequent washes with stain buffer [PBS and 2% fetal calf serum] after surface staining. Cells were then washed twice with Permeabilization Wash Buffer and incubated with intracellular antibodies against IFN-γ BV785 (0.12 µg, clone: KMG1.1, Biolegend, catalog number 554412), IL-17 APC (0.12 µg, clone: Bio17B, ebioscience, catalog number 505838) and Foxp3 PE Cy7 (0.06 µg, clone: FJK-16s, Invitrogen, catalog number 17-6988-82) for 45 min on ice. Counting beads (25,000 beads) were added to every sample to normalize the total number of cells. Flow cytometry was performed on an LSRII or Fortessa (BD Biosciences) cytometer and analyzed with FlowJo software.

### Data analysis

Data were compared using one-way analysis of variance (ANOVA) with the Tukey post-test, or two-way ANOVA with repeated measures and the Tukey post-test, where appropriate. For cell numbers and bacterial CFU, values were log_10_-transformed prior to analysis. Differences were considered significant when *p* < 0.05. All data were analyzed using GraphPad Prism.

### Reporting summary

Further information on research design is available in the Nature Research Reporting Summary linked to this article.

## Supplementary information

Supplement

REPORTING SUMMARY

## Data Availability

All data generated or analyzed during this study are presented in the article, and materials are available from the corresponding author upon reasonable request.
