# Supplementary material for: Toxin expression during Staphylococcus aureus infection imprints host immunity to inhibit vaccine efficacy
Source: NPJ Vaccines. 2023 Jan 24;8:3. doi: 10.1038/s41541-022-00598-3 (PMC9873725; doi:10.1038/s41541-022-00598-3)
Supplement: Supplementary file 1 — Supplement [file 41541_2022_598_MOESM1_ESM.pdf]

SUPPLEMENTARY FIGURES

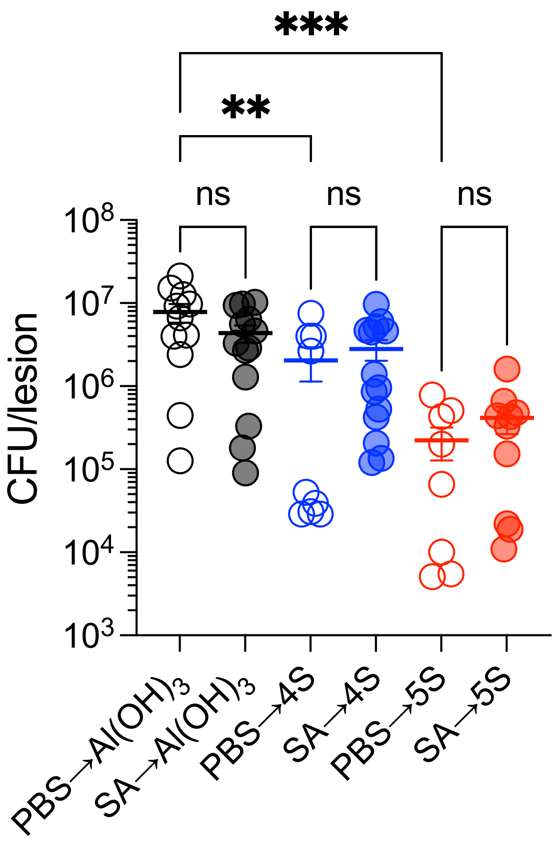

Supplementary Figure 1. Impact of *S. aureus* SSTI on vaccine-mediated bacterial clearance from skin lesions. Experimental model depicted in Fig 1: C57BL/6 mice were infected with *S. aureus* (SA) SSTI followed by vaccination with "4S" (LukE, LukS-PV, SplB, SspB) or "5S" (4S + HlaH35L), followed by secondary SSTI. Bacterial CFU from the skin lesions 7d after secondary SSTI. N=9-14 mice/group, pooled from 2 experiments. Data are presented as mean ± SEM and were analyzed using 1-way ANOVA with Tukey's post-test. ** $p< 0.01$; *** $p<0.001$; NS not significant.

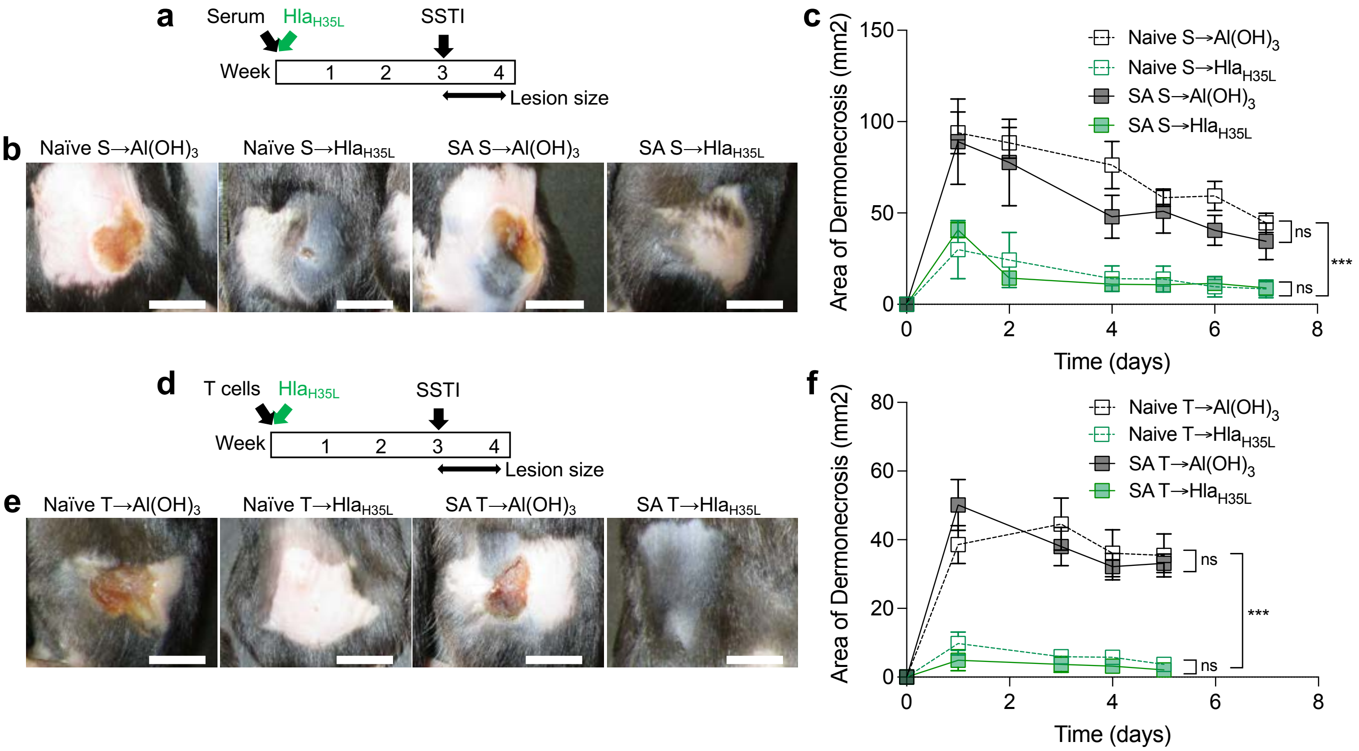

Supplementary Figure 2. Vaccine inhibition is not transferrable by serum or T cells. (A,D) Experimental models: serum (A) or T cells (D) from convalescent C57BL/6 mice (following *S. aureus* SSTI) were transferred to naïve mice one day prior Hla_{H35L} vaccination. Three weeks after vaccination, the mice received secondary *S. aureus* SSTI. (B,E) Representative photos of mouse lesions on day 2. Scale bar = 10 mm. (B,C) There were no differences in vaccine-mediated protection against dermonecrosis in mice that received convalescent serum prior to vaccination (SA S→Hla_{H35L}) and mice that received naïve serum (Naïve S→Hla_{H35L}). (E,F) Similarly, there were no differences in vaccine-mediated protection against dermonecrosis in mice that received convalescent T cells prior to vaccination (SA T→Hla_{H35L}) and mice that received naïve T cells (Naïve T→Hla_{H35L}). N=5 mice/group; 1 representative experiment of 2 repeats is presented. Data are presented as mean ± SEM and were analyzed using 2-way ANOVA with repeated measures and Tukey's post-test. *** indicates p<0.001; NS not significant.

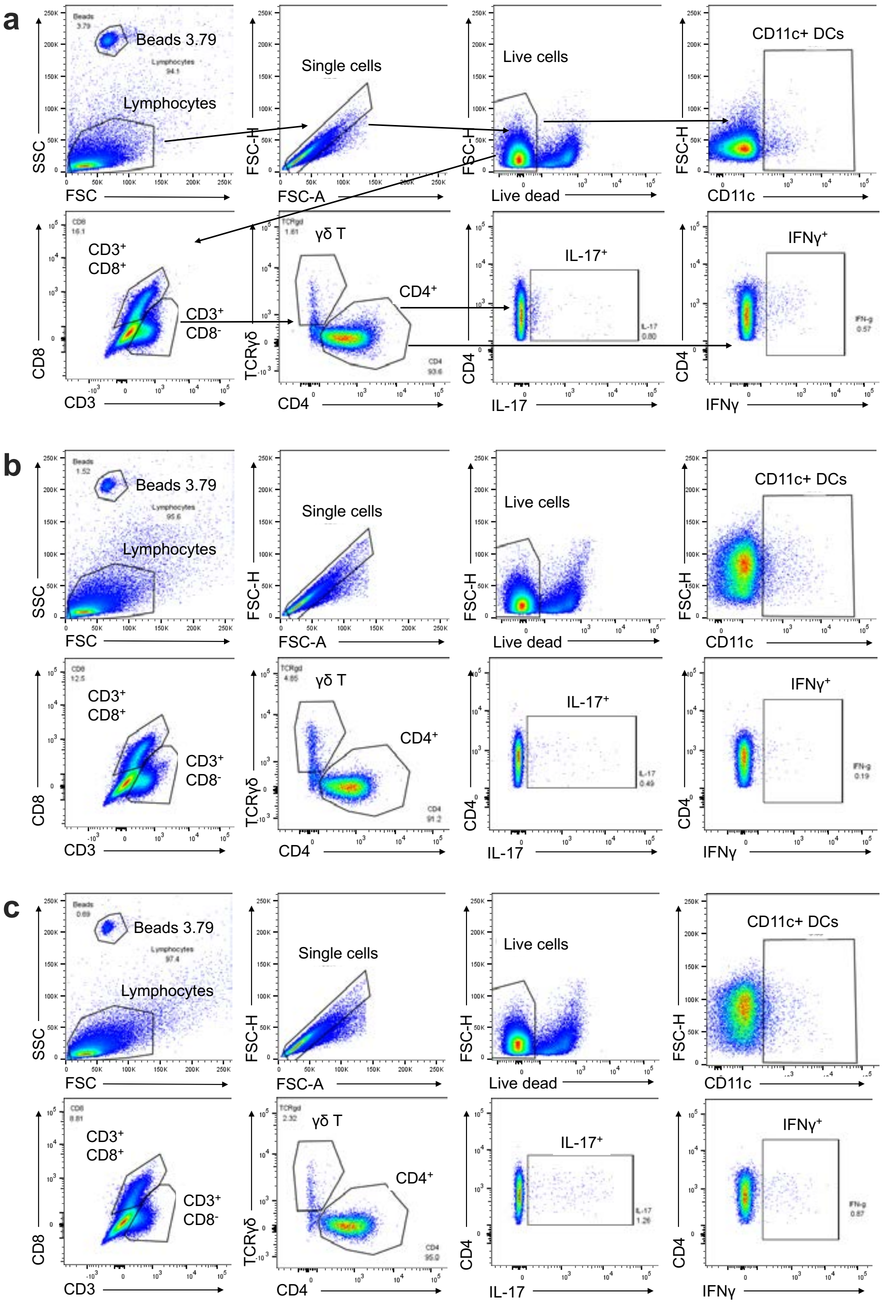

Supplementary Figure 3. Gating strategy for quantification of T cell and dendritic cell (DC) populations. Forward scatter area (FSC-A) versus side scatter area (SSC-A) were used to exclude debris and include cells and counting beads. FSC-A and FSC-H were used to select single cells. FSC-H and APC-Cy7 were used to select live cells and exclude dead cells. CD8-PerCP-Cy5 and CD3-FITC were used to select CD8 and CD3 (no CD8) cells populations. γδ TCR-PE and CD4-BUV396 were used to select γδ TCR and CD4 T cell populations. IFN-γ and IL-17 were quantified by gating positive cells using CD4-BUV396 and IFN-γ-BV785, IL-17-APC. DCs were quantified using CD11c eflur450. Representative flow plot from dLN of mice (1 week post-infection) that received PBS (A), SSTI with wild-type *S. aureus* (B), or SSTI with Δ*hla* (C).

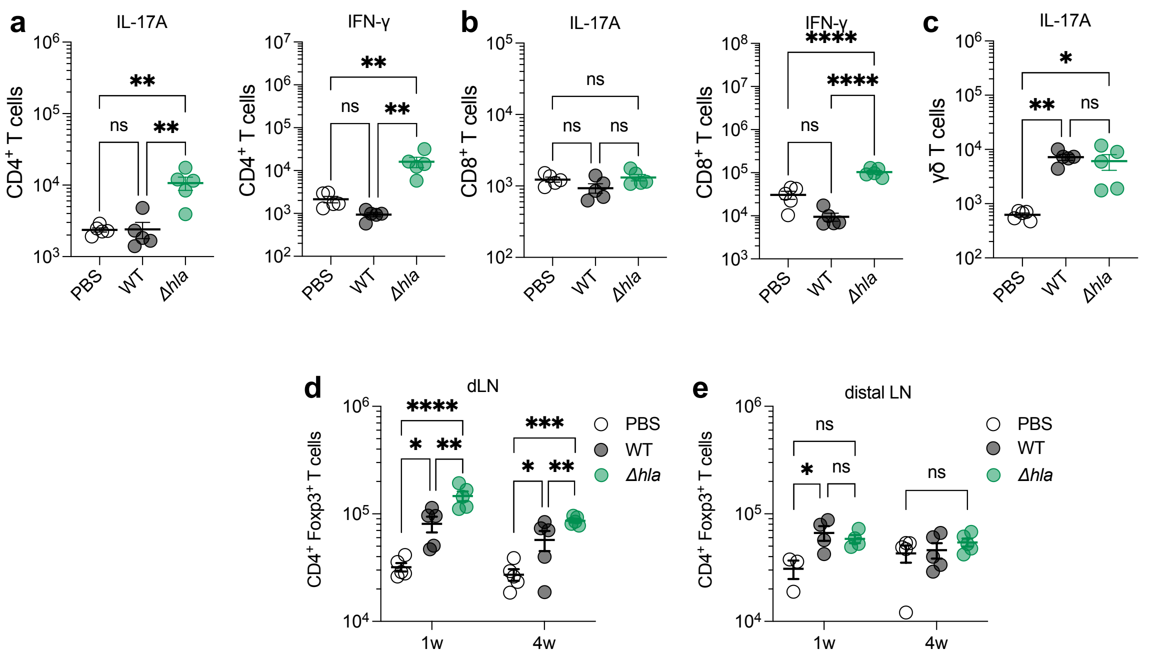

Supplementary Figure 4. Impact of *hla* expression on IL-17A$^+$, IFNγ$^+$ and Foxp3$^+$ T cell populations. (A-B) Following SSTI with wild-type (WT) *S. aureus* or an isogenic *hla* deletion mutant (Δ*hla*), local draining lymph nodes (dLN) or distal LN were harvested 1 and 4 weeks after infection and flow cytometry performed. (A-C) IL-17A$^+$ and IFNγ$^+$ CD4$^+$, CD8$^+$, and γδ T cells were quantified in local dLNs 1 week after SSTI with WT *S. aureus* or Δ*hla*. (A) There were higher numbers of IL-17A$^+$ and IFNγ$^+$ CD4$^+$ T cells following infection with Δ*hla*, compared with WT. (B) There higher numbers of IFNγ$^+$ CD8$^+$ T cells following infection with Δ*hla*, compared with WT, but no significant differences in IL-17A$^+$ CD4$^+$ T cells between the groups. (C) Similarly, there were higher numbers of IFNγ$^+$γδ T cells following infection with Δ*hla*, compared with WT, but no significant differences in IL-17A$^+$ γδ T cells between the groups. (D) There were higher numbers of CD4$^+$ Foxp3$^+$ T cells in local dLNs 1 week following infection with Δ*hla*, compared with WT. These differences persisted 4 weeks after infection. (E) In contrast, there were no significant differences in the numbers of CD4$^+$ Foxp3$^+$ T cells in distal LNs 1 or 4 weeks following infection between mice infected with WT or Δ*hla.* N=5 mice/group; 1 representative experiment of at least 2 repeats is presented. Data are presented as mean ± SEM and were analyzed using 1-way ANOVA on $\log_{10}$-transformed values with Tukey's post-test. * indicates *p*<0.05; ** *p*< 0.01; *** *p*<0.001; **** *p*<0.0001; NS not significant.

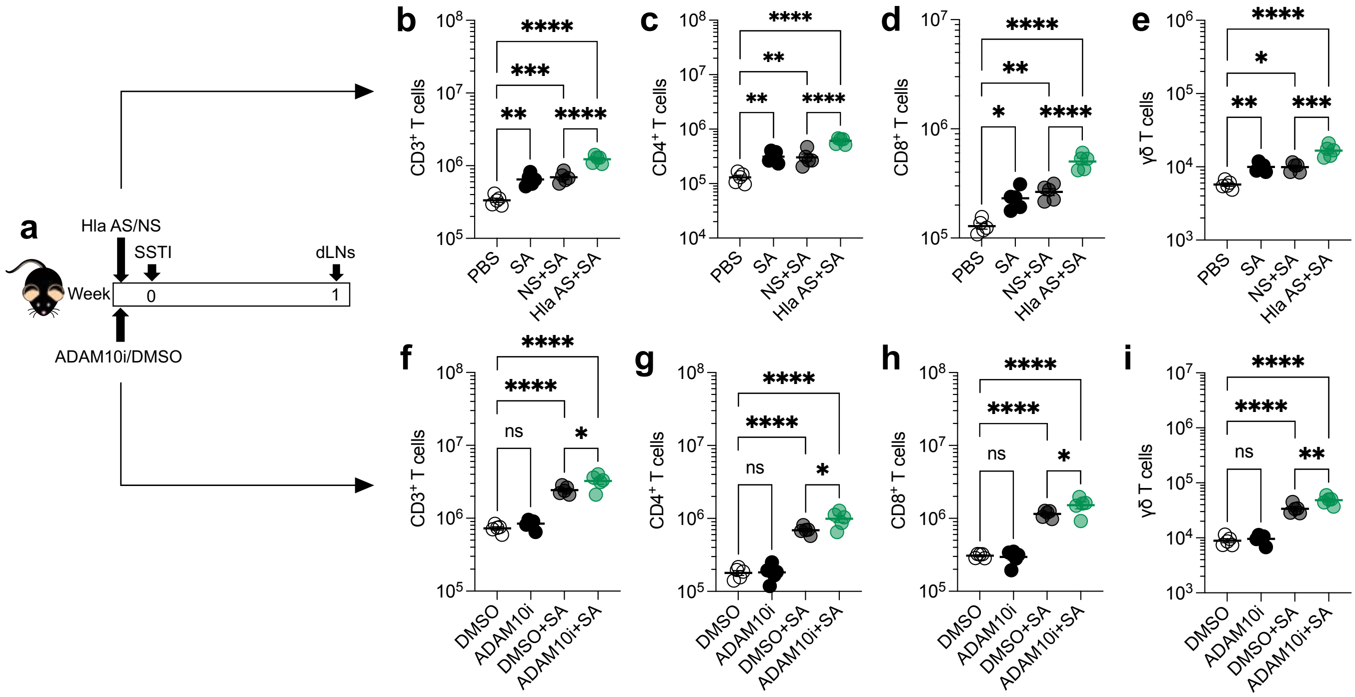

Supplementary Figure 5. Impact of *hla* neutralization and chemical inhibition of ADAM10 on T cell populations in dLNs. (A) Experimental models: 1 day prior to *S. aureus* SSTI, C57BL/6 mice received 200 μl Hla-specific antiserum (Hla AS) or naïve serum (NS) (top) or were treated with the ADAM10 inhibitor (ADAM10i) GI254023X or vehicle alone (DMSO) (bottom). Hla AS was obtained from Hla_{H35L} vaccinated C57BL/6 mice. 7 days following infection, local draining lymph nodes (dLN) were harvested for flow cytometric quantification of $CD3^+$, $CD4^+$, $CD8^+$, γδ T cells. (B-E) There were higher numbers of $CD3^+$, $CD4^+$, $CD8^+$, and γδ T cell populations in local dLNs following SSTI in mice that received Hla-specific antiserum (Hla AS+SA), compared with infected mice that received naïve serum (NS+SA). (F-I) Similarly, there were higher numbers of $CD3^+$, $CD4^+$, $CD8^+$, and γδ T cells in local dLNs in mice that received ADAM10 inhibitor prior to SSTI (ADAM10i+SA), compared with mice that received vehicle alone (DMSO+SA). N=5 mice/group; 1 representative experiment of 2 repeats is presented. Data are presented as mean ± SEM and were analyzed using 1-way ANOVA on log_{10}-transformed values with Tukey's post-test. * indicates $p<0.05$; ** $p<0.01$; *** $p<0.001$; **** $p<0.0001$; NS not significant.

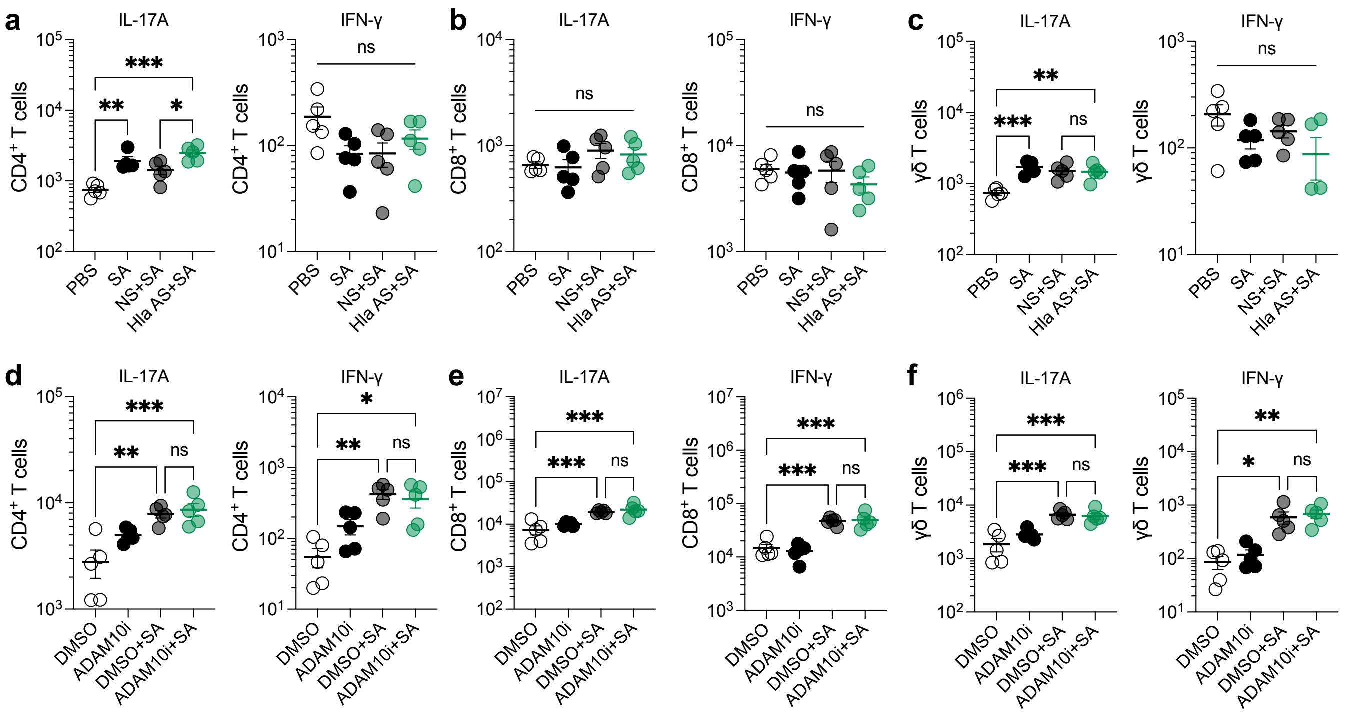

Supplementary Figure 6. Impact of *hla* neutralization and chemical inhibition of ADAM10 on IL-17A$^+$ and IFNγ$^+$ T cell populations in local dLNs. Using the models depicted in Fig S4A, mice received 200μl Hla-specific antiserum (Hla AS) or naïve serum (NS) (top) or were treated with the ADAM10 inhibitor (ADAM10i) GI254023X or vehicle alone (DMSO) (bottom). Hla AS was obtained from Hla_{H35L} vaccinated C57BL/6 mice. 7 days following infection, local draining lymph nodes (dLN) were harvested for flow cytometric quantification of IL-17A$^+$ and IFNγ$^+$ CD4$^+$, CD8$^+$, and γδ T cells. (A) There were higher numbers of IL-17A$^+$ CD4$^+$ T cells in local dLNs in mice that received Hla-specific antiserum prior to SSTI (Hla AS+SA), compared with mice that received naïve serum (NS+SA). However, there were no significant differences in IFNγ$^+$ CD4$^+$ T cells between the groups. There were also no significant differences in IL-17A$^+$ or IFNγ$^+$ CD8$^+$ (B) and γδ T cells (C) between the Hla AS+SA and NS+SA groups. (D-F) There were no significant differences in the numbers of IL-17A$^+$ or IFNγ$^+$ CD4$^+$ (D), CD8$^+$ (E), and γδ T cells (F) between mice that received ADAM10 inhibitor prior to SSTI (ADAM10i+SA) and those that received vehicle alone (DMSO+SA). N=5 mice/group; 1 representative experiment of 2 repeats is presented. Data are presented as mean ± SEM and were analyzed using 1-way ANOVA on log_{10}-transformed values with Tukey's post-test. * indicates *p*<0.05; ** *p*< 0.01; *** *p*<0.001; **** *p*<0.0001; NS not significant.
