# Supplementary material for: Toxin expression during Staphylococcus aureus infection imprints host immunity to inhibit vaccine efficacy
Source: NPJ Vaccines. 2023 Jan 24;8:3. doi: 10.1038/s41541-022-00598-3 (PMC9873725; doi:10.1038/s41541-022-00598-3)
Supplement: Supplementary file 2 — REPORTING SUMMARY [file 41541_2022_598_MOESM2_ESM.pdf]

nature portfolio

Corresponding author(s): Dr Omid Teymournejad

Last updated by author(s): Oct 27, 2022

Reporting Summary

Nature Portfolio wishes to improve the reproducibility of the work that we publish. This form provides structure for consistency and transparency in reporting. For further information on Nature Portfolio policies, see our Editorial Policies and the Editorial Policy Checklist.

Statistics

For all statistical analyses, confirm that the following items are present in the figure legend, table legend, main text, or Methods section.

n/a	Confirmed	
☐	☒	The exact sample size (*n*) for each experimental group/condition, given as a discrete number and unit of measurement
☐	☒	A statement on whether measurements were taken from distinct samples or whether the same sample was measured repeatedly
☐	☒	The statistical test(s) used AND whether they are one- or two-sided<br>*Only common tests should be described solely by name; describe more complex techniques in the Methods section.*
☐	☒	A description of all covariates tested
☐	☒	A description of any assumptions or corrections, such as tests of normality and adjustment for multiple comparisons
☐	☒	A full description of the statistical parameters including central tendency (e.g. means) or other basic estimates (e.g. regression coefficient) AND variation (e.g. standard deviation) or associated estimates of uncertainty (e.g. confidence intervals)
☐	☒	For null hypothesis testing, the test statistic (e.g. *F*, *t*, *r*) with confidence intervals, effect sizes, degrees of freedom and *P* value noted<br>*Give P values as exact values whenever suitable.*
☒	☐	For Bayesian analysis, information on the choice of priors and Markov chain Monte Carlo settings
☒	☐	For hierarchical and complex designs, identification of the appropriate level for tests and full reporting of outcomes
☒	☐	Estimates of effect sizes (e.g. Cohen's *d*, Pearson's *r*), indicating how they were calculated

Our web collection on statistics for biologists contains articles on many of the points above.

Software and code

Policy information about availability of computer code

Data collection	No software was used
Data analysis	FlowJo (version 10.7.1), Prism (version 9)

For manuscripts utilizing custom algorithms or software that are central to the research but not yet described in published literature, software must be made available to editors and reviewers. We strongly encourage code deposition in a community repository (e.g. GitHub). See the Nature Portfolio guidelines for submitting code & software for further information.

Data

Policy information about availability of data

All manuscripts must include a data availability statement. This statement should provide the following information, where applicable:

- Accession codes, unique identifiers, or web links for publicly available datasets
- A description of any restrictions on data availability
- For clinical datasets or third party data, please ensure that the statement adheres to our policy

All data generated or analyzed during this study are presented in the article, and materials are available from the corresponding author upon reasonable request.

Human research participants

Policy information about studies involving human research participants and Sex and Gender in Research.

Reporting on sex and gender	*Use the terms sex (biological attribute) and gender (shaped by social and cultural circumstances) carefully in order to avoid confusing both terms. Indicate if findings apply to only one sex or gender; describe whether sex and gender were considered in study design whether sex and/or gender was determined based on self-reporting or assigned and methods used. Provide in the source data disaggregated sex and gender data where this information has been collected, and consent has been obtained for sharing of individual-level data; provide overall numbers in this Reporting Summary. Please state if this information has not been collected. Report sex- and gender-based analyses where performed, justify reasons for lack of sex- and gender-based analysis.*
Population characteristics	*Describe the covariate-relevant population characteristics of the human research participants (e.g. age, genotypic information, past and current diagnosis and treatment categories). If you filled out the behavioural & social sciences study design questions and have nothing to add here, write "See above."*
Recruitment	*Describe how participants were recruited. Outline any potential self-selection bias or other biases that may be present and how these are likely to impact results.*
Ethics oversight	*Identify the organization(s) that approved the study protocol.*

Note that full information on the approval of the study protocol must also be provided in the manuscript.

Field-specific reporting

Please select the one below that is the best fit for your research. If you are not sure, read the appropriate sections before making your selection.

☒ Life sciences ☐ Behavioural & social sciences ☐ Ecological, evolutionary & environmental sciences

For a reference copy of the document with all sections, see nature.com/documents/nr-reporting-summary-flat.pdf

Life sciences study design

All studies must disclose on these points even when the disclosure is negative.

Sample size	No sample-size calculation was performed.
Data exclusions	No data were excluded from analysis
Replication	Experiments were replicated at least twice and all attempts at replication were successful
Randomization	Not applicable.
Blinding	Investigators were not blinded because it was not feasible (i.e. study groups were immediately apparent).

Reporting for specific materials, systems and methods

We require information from authors about some types of materials, experimental systems and methods used in many studies. Here, indicate whether each material, system or method listed is relevant to your study. If you are not sure if a list item applies to your research, read the appropriate section before selecting a response.

Materials & experimental systems

n/a	Involved in the study	
☐	☒	Antibodies
☒	☐	Eukaryotic cell lines
☒	☐	Palaeontology and archaeology
☐	☒	Animals and other organisms
☒	☐	Clinical data
☒	☐	Dual use research of concern

Methods

n/a	Involved in the study	
☒	☐	ChIP-seq
☐	☒	Flow cytometry
☒	☐	MRI-based neuroimaging

Antibodies

Antibodies used	For surface staining, antibodies against CD8 PerCP Cy 5.5 (53-6.7), CD3 FITC (145-2C11), CD4 BUV395 (GK1.5), CD11c eflur450 (N418), and γδ TCR PE (GL3) were used. Intracellular antibodies against IFN-γ BV785 (KMG1.1), IL-17 APC (Bio17B) and Foxp3 PE Cy7 (FJK-16s). Antibodies were purchased from eBioscience, BioLegend, Invitrogen, or Becton Dickinson
Validation	Validation statements are provided on the manufacture's website

Animals and other research organisms

Policy information about studies involving animals; ARRIVE guidelines recommended for reporting animal research, and Sex and Gender in Research

Laboratory animals	Female C57BL/6 mice were purchased from Taconic. Primary infection or vaccination was performed when the mice were 7 to 8 weeks old.
Wild animals	The study did not involve wild animals
Reporting on sex	We have optimized our models using female C57BL/6 mice from Taconic, because we have found that male mice are difficult to house communally for our longer-term studies (i.e. 12-16 weeks). Specifically, in the SSTI model, long-term communal housing results in local skin irritation and inflammation that inconsistently impacts lesion severity and immune responses during secondary infection.
Field-collected samples	The study did not involve samples collected from the field.
Ethics oversight	All animal experiments were approved by the Institutional Animal Care and Use Committee (IACUC) at the Abigail Wexner Research Institute at Nationwide Children's Hospital (protocol no. AR17-00072) and adhered to the standards of NIH Guide for the Care and Use of Laboratory Animals.

Note that full information on the approval of the study protocol must also be provided in the manuscript.

Flow Cytometry

Plots

Confirm that:

- [x] The axis labels state the marker and fluorochrome used (e.g. CD4-FITC).
- [x] The axis scales are clearly visible. Include numbers along axes only for bottom left plot of group (a 'group' is an analysis of identical markers).
- [x] All plots are contour plots with outliers or pseudocolor plots.
- [x] A numerical value for number of cells or percentage (with statistics) is provided.

Methodology

Sample preparation	Lymphocytes were isolated from draining lymph nodes following SSTI and processed into single-cell suspensions. Cells were stained for flow cytometry using AquaFluor LiveDead (Life Technologies) solution to exclude dead cells. Two different panels were used for surface and intracellular staining. For surface staining, antibodies against CD8 PerCP Cy 5.5 (53-6.7), CD3 FITC (145-2C11), CD4 BUV395 (GK1.5), CD11c eflur450 (N418), and γδ TCR PE (GL3) were used. Cells were incubated with antibodies for 30 minutes on ice in the dark. For intracellular staining, cells were fixed and permeabilized using Fixing/ Permeablization solution for 30 min in the dark at RT following two subsequent washes with stain buffer [PBS and 2% fetal calf serum (FCS)] after surface staining. Cells were then washed twice with Permeabilization Wash Buffer and incubated with intracellular antibodies against IFN-γ BV785 (KMG1.1), IL-17 APC (Bio17B) and Foxp3 PE Cy7 (FJK-16s) for 45 minutes on ice. Counting beads (25,000 beads) were added to every sample to normalize the total number of cells. Antibodies were purchased from eBioscience, BioLegend, Invitrogen, or Becton Dickinson.
Instrument	Flow cytometry was performed on an LSRII or Fortessa (BD Biosciences) cytometer
Software	Analyzed with FlowJo software
Cell population abundance	we started with 1-3 million cells per group before staining and measured 100,000 -200,000 cells with FACS machine
Gating strategy	Gating strategy for quantification of T cell and dendritic cell (DC) populations. Forward scatter area (FSC-A) versus side scatter area (SSC-A) were used to exclude debris and include cells and counting beads. FSC-A and FSC-H were used to select single cells. FSC-H and APC-Cy7 were used to select live cells and exclude dead cells. CD8-PerCP-Cy5 and CD3-FITC were used to select CD8 and CD3 (no CD8) cells populations. γδ TCR-PE and CD4-BUV396 were used to select γδ TCR and CD4 T cell populations. IFN-γ and IL-17 were quantified by gating positive cells using CD4-BUV396 and IFN-γ-BV785, IL-17-APC. DCs were quantified using CD11c eflur450.

- [x] Tick this box to confirm that a figure exemplifying the gating strategy is provided in the Supplementary Information.
